# Mapping DNA interaction landscapes in psoriasis susceptibility loci highlights *KLF4* as a target gene in 9q31

**DOI:** 10.1186/s12915-020-00779-3

**Published:** 2020-05-04

**Authors:** Helen Ray-Jones, Kate Duffus, Amanda McGovern, Paul Martin, Chenfu Shi, Jenny Hankinson, Oliver Gough, Annie Yarwood, Andrew P. Morris, Antony Adamson, Christopher Taylor, James Ding, Vasanthi Priyadarshini Gaddi, Yao Fu, Patrick Gaffney, Gisela Orozco, Richard B. Warren, Steve Eyre

**Affiliations:** 1 Centre for Genetics and Genomics Versus Arthritis, Division of Musculoskeletal and Dermatological Sciences, School of Biological Sciences, Faculty of Biology, Medicine and Health, The University of Manchester, Manchester, UK; 2 Dermatology Centre, Manchester NIHR Biomedical Research Centre, Manchester Academic Health Science Centre, Salford Royal NHS Foundation Trust, Manchester, UK; 3 The Lydia Becker Institute of Immunology and Inflammation, The University of Manchester, Manchester, UK; 4 Genome Editing Unit, Faculty of Biology, Medicine and Health, The University of Manchester, Manchester, UK; 5 Genes and Human Disease Research Program, Oklahoma Medical Research Foundation, Oklahoma City, OK 73104 USA

**Keywords:** Psoriasis, Chromatin, GWAS, CHi-C, HiChIP, CRISPR, KLF4

## Abstract

**Background:**

Genome-wide association studies (GWAS) have uncovered many genetic risk loci for psoriasis, yet many remain uncharacterised in terms of the causal gene and their biological mechanism in disease. This is largely a result of the findings that over 90% of GWAS variants map outside of protein-coding DNA and instead are enriched in cell type- and stimulation-specific gene regulatory regions.

**Results:**

Here, we use a disease-focused Capture Hi-C (CHi-C) experiment to link psoriasis-associated variants with their target genes in psoriasis-relevant cell lines (HaCaT keratinocytes and My-La CD8+ T cells). We confirm previously assigned genes, suggest novel candidates and provide evidence for complexity at psoriasis GWAS loci. For one locus, uniquely, we combine further epigenomic evidence to demonstrate how a psoriasis-associated region forms a functional interaction with the distant (> 500 kb) *KLF4* gene. This interaction occurs between the gene and active enhancers in HaCaT cells, but not in My-La cells. We go on to investigate this long-distance interaction further with Cas9 fusion protein-mediated chromatin modification (CRISPR activation) coupled with RNA-seq, demonstrating how activation of the psoriasis-associated enhancer upregulates *KLF4* and its downstream targets, relevant to skin cells and apoptosis.

**Conclusions:**

This approach utilises multiple functional genomic techniques to follow up GWAS-associated variants implicating relevant cell types and causal genes in each locus; these are vital next steps for the translation of genetic findings into clinical benefit.

## Background

Psoriasis is a common immune-mediated disease that causes the formation of red, scaly plaques on the skin. On a cellular basis, psoriasis is thought to be driven by a complex interplay between the immune system, such as T cells and dendritic cells, and the resident cells of the epidermis. Keratinocytes are the most abundant cell type in the epidermis [1] and are highly dysregulated in psoriasis, undergoing accelerated differentiation and migration to the surface of the skin where they create the characteristic plaques [2]. T lymphocytes, such as CD4+ and CD8+, infiltrate the epidermis and release pro-inflammatory cytokines such as IL-17, IFN-γ and IL-22 [3–5]. Keratinocytes respond to immune signals and release chemokines that can in turn recruit T cells and dendritic cells; this feedback loop leads to prolonged inflammation [4, 6].

Genetic predisposition is the largest risk factor for psoriasis. Genome-wide association studies (GWAS) have so far identified 63 loci associated with susceptibility in individuals of European ancestry [7] and more than 20 further unique signals in the Han Chinese population [8, 9]. The majority of single nucleotide polymorphisms (SNPs) associated with psoriasis and other immune-mediated conditions do not map within gene coding regions; rather, they are enriched in non-coding enhancer elements [10], with approximately 60% of predicted causal SNPs lying within cell type-specific immune enhancers relevant to the disease of interest and approximately 8% in promoters [11, 12]. Historically, gene candidates were assigned to GWAS loci based on proximity or biological function; however, this can lead to incorrect interpretation of results since it is now well established that enhancers can regulate genes over very large genomic distances through chromatin looping [13, 14].

The challenge now is to link disease-associated enhancers with the true genes that they regulate in order to determine the relevant cell types and the mechanism of regulation. Advances in sequencing, molecular biology and genome editing are now enabling us to answer these pivotal, “post-GWAS” questions [15]. Hi-C is a technique used to map interactions between distant DNA elements [14, 16]. Its more recent derivative, Capture Hi-C (CHi-C), allows for high-depth characterisation of DNA interactions in loci of interest [17]. CHi-C has been applied to gene promoters in multiple blood cell types [13] and to GWAS loci in diseases such as cancer [17–19] and autoimmune conditions [20]. HiChIP builds on Hi-C by enriching for interactions that colocalise with an immunoprecipitated chromatin fraction (such as that marked by histone 3 lysine 27 acetylation, a hallmark of active chromatin) [21]. HiChIP was recently applied in primary T cells [22] and B cells, in the context of systemic lupus erythematosus (SLE) [23]. To empirically determine the function of GWAS SNPs, direct perturbation is now widely carried out using the CRISPR-Cas9 system, either through genome editing or chromatin activation/interference (CRISPRa/CRISPRi) of promoters and enhancers [24].

Here, we apply these technologies to annotate gene targets within known psoriasis GWAS loci utilising two relevant human cell lines: HaCaT (keratinocytes) and My-La (CD8+ T cells). We go on to experimentally characterise an intergenic psoriasis risk locus where the gene candidate, Krüppel-like factor 4 (*KLF4*), encodes a transcription factor with a range of relevant functions including skin barrier formation and immune signalling, but is situated more than 500 kb from the lead GWAS SNP. This study provides further evidence that *KLF4* is a distal gene target in 9q31.2.

## Results

### Capture Hi-C identified novel gene targets in psoriasis susceptibility loci

We generated sequencing data for region CHi-C experiments in duplicate in three conditions: (i) HaCaT unstimulated, (ii) HaCaT stimulated with IFN-γ to represent the inflammatory psoriatic environment and (iii) My-La cells. Our overarching design targeted genetic regions associated with several immune-mediated diseases including psoriasis SNPs from multiple GWAS (see the “Methods” and Additional file 1, Table S1). We aimed for 10,000 and obtained an average of 8580 mapped Hi-C fragments (di-tags) per bait fragment with a mean capture efficiency of 71% (Additional file 1, Table S2). Capture Hi-C Analysis of Genomic Organisation (CHiCAGO) was used to identify significant interactions for each cell type. Reproducibility was assessed firstly by observing the number of shared interactions between replicates and secondly through HiCRep [25] (Additional file 2, Fig. S1). The stratum-adjusted correlation coefficient (SCC) produced by HiCRep showed that all HaCaT samples were highly similar and were slightly more correlated by replicate rather than by condition. My-La samples were also highly correlated with each other, and less so with HaCaT cells (Additional file 2, Fig. S1B).

By integrating published ChIP-seq data, we found that other-end fragments interacting with the GWAS bait fragments were enriched in H3K27ac and H3K4me3 in related cell types NHEK and CD8+ naive T cells (Additional file 3, Fig. S2), suggesting that the GWAS loci preferentially interact with active regions such as enhancers and promoters. Other-end fragments in HaCaT cells were also enriched for the chromatin structural regulator CTCF, based on ChIP-seq in NHEK (Additional file 3, Fig. S2).

For the My-La cell line, we noted a large number of significant *trans*-interactions (CHiCAGO score ≥ 5) spanning different chromosomes (3392/42,928 total interactions from all captured immune-mediated disease loci), and the majority of these (~ 59%) mapped to interactions between two known translocated loci in My-La cells [26]. In light of this, the interactions were filtered to only include same-chromosome interactions. We then filtered the CHi-C interactions to include only those involving psoriasis GWAS loci; we had successfully targeted 104 lead GWAS SNPs at genome-wide significance and their associated proxy SNPs at *r*^2^ > 0.8, corresponding to 907 HindIII bait fragments. Across the three capture experiments, we obtained an average of 6593 interactions (CHiCAGO score ≥ 5) originating from targeted psoriasis fragments (Additional file 1, Table S2). The data were enriched for long-range interactions, with more than 75% of the significant interactions in the psoriasis loci spanning > 100 kb (Additional file 4, Fig. S3). The median interaction distances were 227 kb (HaCaT unstimulated), 234 kb (HaCaT stimulated) and 259 kb (My-La). The interaction distances in psoriasis loci were found to be significantly greater in My-La cells than in HaCaT cells (Kruskal-Wallis with Dunn’s multiple comparisons test; *P* < 0.0001 and *P* = 0.0011 for HaCaT unstimulated and stimulated, respectively), suggesting cell-specific chromatin architecture.

To validate our CHi-C data, we overlaid the interactions with a published expression quantitative trail locus (eQTL) dataset, in which the lead psoriasis SNP had been colocalised with the lead eQTL SNP in CD4+ T cells and monocytes [27]. We hypothesised that long-distance eQTL-gene promoter pairings would often implicate chromatin looping. The study reported 15 lead GWAS SNPs with 26 corresponding lead eQTL proxy SNPs, of which 16 proxies, representing 9 lead GWAS SNPs, overlapped baited fragments in our study. Eight of these proxies were captured within a HindIII fragment that contained, or was within 20 kb of, the eQTL gene itself. A further seven proxies were within, or adjacent to, fragments that showed evidence of interacting with the distal eQTL gene in our cell line CHi-C data (CHiCAGO score ≥ 5) (Additional file 5, Table S3). Only the most distant proxy, rs8060857, did not show any evidence of interacting with the eQTL gene (*ZNF750*, 720 kb). Therefore, this is a strong evidence that our CHi-C data can show links between distal functional GWAS SNPs and their target gene, even across non-matched cell types.

In all the cell lines, approximately 30% of the interactions occurred between the psoriasis bait fragment and a transcription start site (Ensembl 75). The total number of interacting gene targets was 442 in unstimulated HaCaT cells (Additional file 5, Table S4), 461 in stimulated HaCaT cells (Additional file 5, Table S5) and 650 in My-La cells (Additional file 5, Table S6), comprising a set of 839 genes. Of these, 288 gene targets (34.3%) were shared between all cell types, whilst 58, 64 and 291 targets were unique in unstimulated HaCaT, IFN-γ-stimulated HaCaT and My-La cells, respectively. Unstimulated and stimulated HaCaT cells shared a large proportion of their gene targets (355 targets; 77–80%). Bait fragments that interacted with genes tended to interact with multiple promoter-containing fragments corresponding to different genes, a median of 2 fragments in HaCaT cells (unstimulated or stimulated) and 3 fragments in My-La cells, implicating between 2 and 3 genes (Additional file 6, Fig. S4), which is in line with previously reported findings [20, 22, 28].

We reasoned that gene targets with detectable expression in the same cell type would be more biologically relevant than those not expressed, so we performed RNA-seq (Additional file 7, Table S7) and determined the relative expression of genes interacting with psoriasis GWAS SNPs in each cell type (Additional file 5, Tables S4-S6) or overlapping GWAS bait fragments (Additional file 7, Table S8). Expressed genes interacting with GWAS fragments included compelling psoriasis candidates such as *IL23A*, *PTGER4*, *STAT3* and *NFKBIZ*. Importantly, we found that other-end fragments of CHi-C interactions were significantly enriched for transcription start sites of expressed genes in the corresponding cell type (Additional file 3, Fig. S2). We searched for transcription factor binding motifs intersected by psoriasis SNPs interacting with active gene promoters using the tool SNP2TFBS [29] and discovered several significantly enriched factors, with the greatest enrichment found for REL, which is itself a candidate gene in the 2p16.1 locus [30] (Additional file 7, Table S9).

Stimulating HaCaT cells with IFN-γ caused the differential expression of 535 genes (adjusted *P* < 0.10): 88 downregulated and 447 upregulated (Additional file 7, Table S10). Whilst the downregulated genes were not enriched for any biological pathways, the upregulated genes were enriched for 196 biological processes that included psoriasis-relevant GO terms such as “GO:0045087 innate immune response” (*P* = 9.39 × 10^−20^), “GO:0034097 response to cytokine stimulus” (*P* = 7.32 × 10^−15^) and “GO:0034340 response to type I interferon” (*P* = 1.08 × 10^−10^) (Additional file 7, Table S11). Twelve of the differentially expressed genes overlapped a psoriasis capture bait fragment (Additional file 3, Table S11) and included *ERAP1*, *ERAP2*, *IFIH1*, *RNF114*, *SOCS1* and *STAT2*. In addition, 12 differentially expressed genes were involved in bait-promoter interactions (Additional file 7, Table S12) and included candidates such as *ICAM1*, *KLF4* and *STAT3*. However, the vast majority of these differentially expressed genes interacted similarly with the psoriasis-associated baits in both unstimulated and stimulated cells (CHiCAGO score ≥ 5).

### Examples of CHi-C interactions implicating target genes for psoriasis

**Fig. 1:**
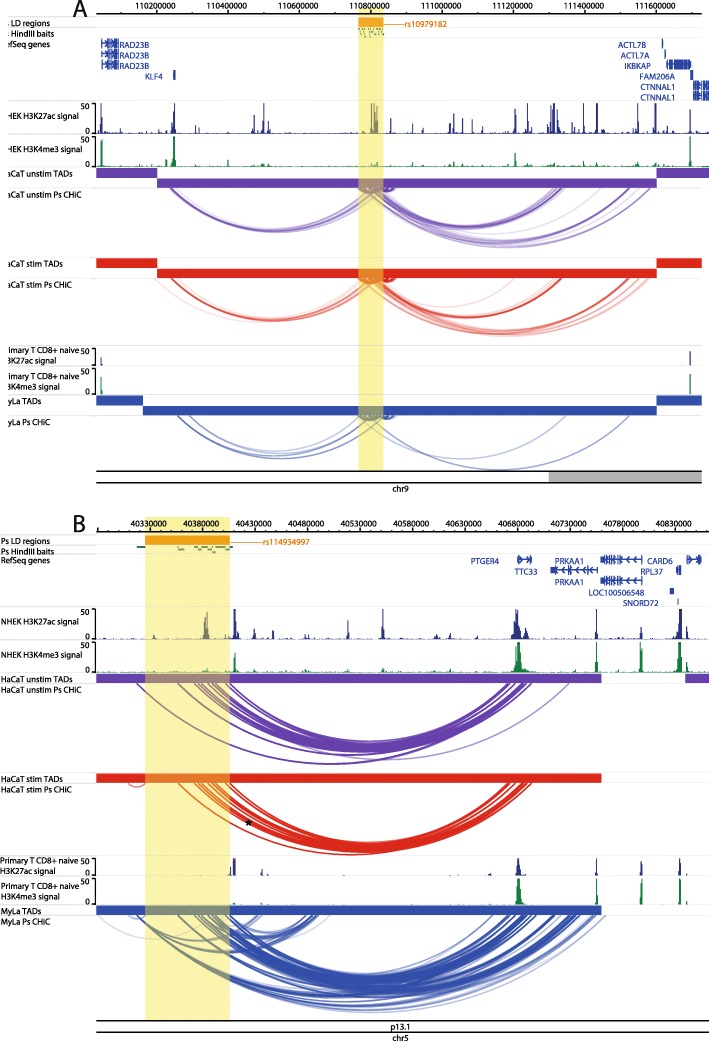
Examples of CHi-C Interactions implicating nearest/reported genes. Interactions are shown in the 9q31.2 (*KLF4*) locus (**a**) and the 5p13.1 (*PTGER4*) locus (**b**). The tracks include psoriasis (Ps) LD blocks as defined by SNPs in *r*^2^ > 0.8 with the index SNP, baited HindIII fragments, RefSeq genes (NCBI), H3K27ac and H3K4me3 *P* value signal in NHEK (ENCODE) and CD8+ primary naive T cells (Roadmap Epigenomics), TADs (shown as bars) and CHi-C interactions significant at CHiCAGO score ≥ 5 (shown as arcs) in three conditions: unstimulated HaCat cells (purple), HaCaT cells stimulated with IFN-γ (red) and My-La cells (blue). The highlighted region indicates the psoriasis LD block. The figure was made with the WashU Epigenome Browser, GRCh37/hg19 [31]

At the intergenic locus 9q31.2, the psoriasis association falls between two distant gene clusters where the suggested gene candidate was Krüppel-like factor 4 (*KLF4*) due to its relevant biological functions in differentiation and innate immunity [30]. Our CHi-C data showed significant interactions (CHiCAGO score ≥ 5) between the psoriasis association and the promoter of *KLF4* in both unstimulated and stimulated HaCaT cells, over a distance of approximately 560 kb (Fig. 1a). In both conditions, the bait fragment chr9:110810592-110816598 interacted with *KLF4* (CHiCAGO score = 6.75 and 5.29 for unstimulated and stimulated cells, respectively) whilst in stimulated cells alone, a second bait fragment (chr9:110798319-110798738) also interacted, coinciding with a more than fivefold increase in the *KLF4* expression (FC = 5.78; adj. *P* = 4.26 × 10^−8^). In My-La cells, a similar conformation was observed; however, the interactions did not coincide with the fragment containing the gene itself. Furthermore, the *KLF4* expression was not detected by RNA-seq in My-La cells, suggesting a cell type-specific mechanism (Additional file 7, Table S7). In all cell types, long-range interactions also stretched from the psoriasis locus to the telomeric side of the gene desert but fell short of the nearest gene on that side (*ACTL7B*) by approximately 35 kb.

At the 5p13.1 locus, the psoriasis SNPs are similarly intergenic [32], but the nearest gene *PTGER4* has been shown to be a strong candidate for other autoimmune diseases at this locus [22]. Our CHi-C data showed interactions (CHiCAGO score ≥ 5) between multiple psoriasis-associated fragments and *PTGER4* over approximately 300 kb to the other end of the TAD, a finding that was robust in all cell types (Fig. 1b). *PTGER4* expression was detected by RNA-seq in all cell types (Additional file 7, Table S7). In My-La cells, interactions also stretched to the promoters of *TTC33*, which was expressed in all cell types, and *RPL37*, for which expression was not detected in any cell type.

**Fig. 2:**
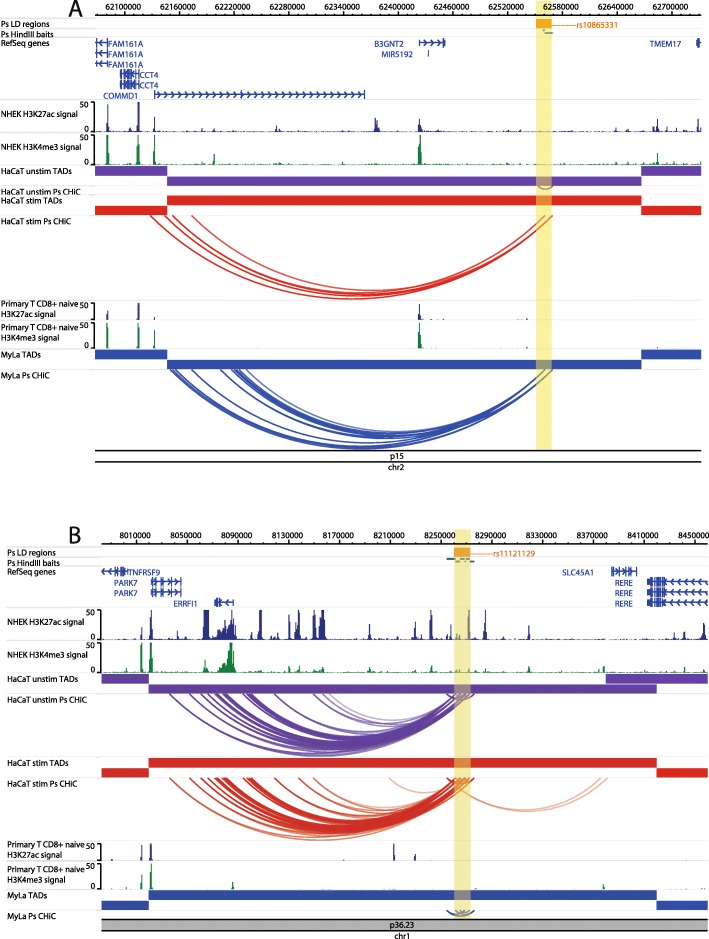
Examples of CHi-C interactions in gene deserts implicating distal/novel genes. Interactions are shown in the 2p15 (*B3GNT2*) locus (**a**) and the 1p36.23 (*RERE*, *SLC45A1*, *ERRFI1*, *TNFRSF9*) locus (**b**). The tracks include psoriasis (Ps) LD blocks as defined by SNPs in *r*^2^ > 0.8 with the index SNP, baited HindIII fragments, RefSeq genes (NCBI), H3K27ac and H3K4me3 *P* value signal in NHEK (ENCODE) and CD8+ primary naive T cells (Roadmap Epigenomics), TADs (shown as bars) and CHi-C interactions significant at CHiCAGO score ≥ 5 (shown as arcs) in three conditions: unstimulated HaCat cells (purple), HaCaT cells stimulated with IFN-γ (red) and My-La cells (blue). The highlighted region indicates the psoriasis LD block. The figure was made with the WashU Epigenome Browser, GRCh37/hg19 [31]

At the 2p15 locus, the psoriasis association tagged by rs10865331 was originally assigned to the nearest gene *B3GNT2*; however, the CHi-C interactions skipped *B3GNT2* (~ 120 kb upstream) and instead implicated the promoter of copper metabolism domain containing 1 (*COMMD1*), a gene involved in NFkB signalling, over approximately 435 kb upstream (Fig. 2a) [30, 33]. This interaction occurred in stimulated HaCaT cells and My-La cells, and *COMMD1* expression was detected by RNA-seq in all cell types. *B3GNT2* expression was also detected in all cell types (Additional file 7, Table S7).

At the 1p36.23 locus, the association tagged by rs11121129 is closest to *SLC45A1* and was originally assigned to multiple putative gene targets [30]. However, the CHi-C data showed interactions (CHiCAGO score ≥ 5) between the psoriasis LD block and the promoter of ERBB receptor feedback inhibitor 1 (*ERRFI1*), an important regulator of keratinocyte proliferation and differentiation, in both unstimulated and stimulated HaCaT cells (Fig. 2b). This interaction was not observed in My-La cells, and moreover, *ERRFI1* expression was detected in HaCaT cells (unstimulated and stimulated) but not in My-La cells (Additional file 7, Table S7). An interaction between the psoriasis association and the promoter of *SLC45A1* was also observed in stimulated, but not in unstimulated, HaCaT cells (Fig. 2b); however, *SLC45A1* expression was not detected by RNA-seq in any of the cell lines (Additional file 5, Table S5).

**Fig. 3:**
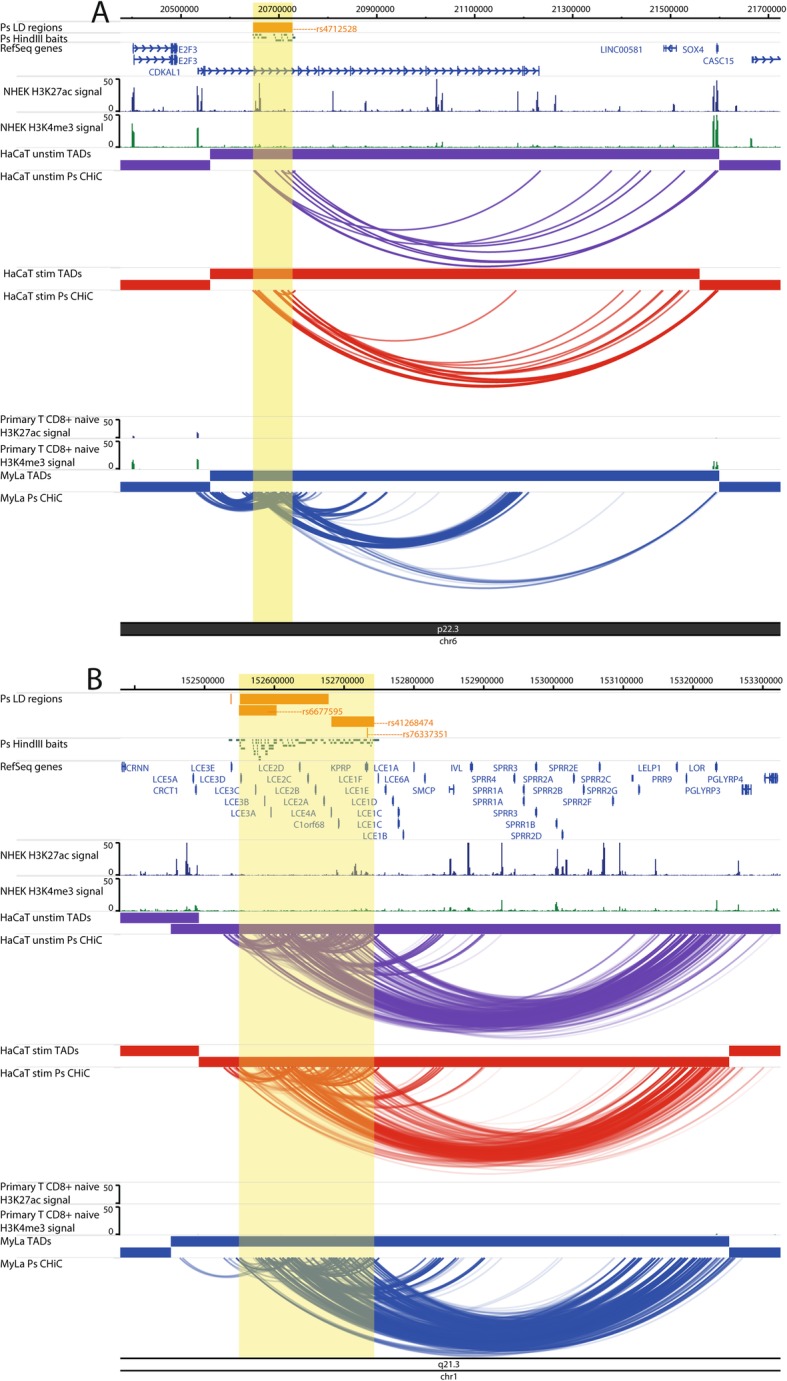
Examples of CHi-C interactions adding complexity to a locus. Interactions are shown in the 6p22.3 (*CDKAL1*) locus (**a**) and the 1q21.3 (*LCE3B*, *LCE3C*) locus (**b**). The tracks include psoriasis (Ps) LD blocks as defined by SNPs in *r*^2^ > 0.8 with the index SNP, baited HindIII fragments, RefSeq genes (NCBI), H3K27ac and H3K4me3 *P* value signal in NHEK (ENCODE) and CD8+ primary naive T cells (Roadmap Epigenomics), TADs (shown as bars) and CHi-C interactions significant at CHiCAGO score ≥ 5 (shown as arcs) in three conditions: unstimulated HaCat cells (purple), HaCaT cells stimulated with IFN-γ (red) and My-La cells (blue). The highlighted region indicates the psoriasis LD block. The figure was made with the WashU Epigenome Browser, GRCh37/hg19 [31]

At the 6p22.3 locus, the psoriasis signal tagged by rs4712528 is intronic to *CDKAL1*, and there were 11 psoriasis-associated intronic fragments that also interacted with the *CDKAL1* promoter in My-La cells (Fig. 3a); *CDKAL1* expression was detected in all cells. However, there were also long-range interactions (CHiCAGO score ≥ 5) between psoriasis-associated fragments and *SOX4* over 950 kb in all cell types (Fig. 3a). *SOX4* is a compelling gene candidate with roles in IL17A production and skin inflammation in mice [34]; here, *SOX4* expression was detected in HaCaT cells but not in My-La cells (Additional file 7, Table S7).

At the 1q21.3 locus, multiple risk SNPs are located at the late cornfield envelope (LCE) gene cluster in the epidermal differentiation complex (EDC). One of the associations in this locus is a 32-kb deletion that removes the *LCE3B* and *LCE3C* genes [30, 35, 36]. The CHi-C data showed multiple, robust interactions between the psoriasis-associated regions at the LCE genes, including from within the 32-kb LCE3C/B-del region, and genes downstream in the EDC that included *IVL*, *LOR*, *PRR9* and *SPRR* genes, over a distance of ~ 600 kb (Fig. 3b). Of these genes, *IVL* interacted with psoriasis baits in unstimulated but not in stimulated HaCaT cells, and its expression decreased upon stimulation (FC = 0.40; adj. *P* = 0.0139). The coding genes directly interacting with fragments within the 32-kb deletion were *LCE3A*, *PRR9*, *LELP1*, *SPRR2B* and *SPRR2C*. Of these, only the expression of proline-rich region 9 (*PRR9*) was detected, in HaCaT cells but not in My-La cells (Additional file 7, Table S7). *PRR9* was previously shown to be upregulated in psoriatic plaques and induced by IL17A and so may be an important distal gene target in this locus [37].

### The 9q31.2 psoriasis risk locus forms long-range interactions with KLF4 and harbours likely regulatory variants

We focused our attention on the large intergenic locus at 9q31.2, which has not previously been characterised in psoriasis, to our knowledge. The candidate gene, Krüppel-like factor 4 (*KLF4*), encodes a transcription factor with a range of relevant functions including skin barrier formation [38] and immune signalling [39] but is situated more than 500 kb from the lead GWAS SNP rs10979182 [30]. The CHi-C experiment showed long-range interactions between the psoriasis-associated SNPs and *KLF4* (Fig. 1a) [30]. *KLF4* expression was also upregulated by IFN-γ, suggesting that it may be an important player within an inflammatory environment. We wanted to prioritise regulatory variants in 9q31.2 and determine if any functional enhancer-promoter relationship existed between the SNPs and *KLF4* or other, distal, genes in the locus.

**Fig. 4:**
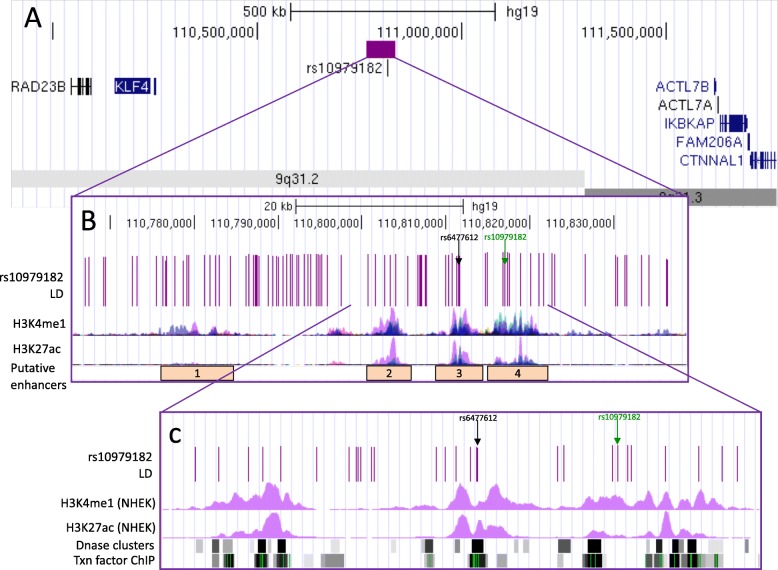
Overview of SNPs in LD with rs10979182 overlaying four putative enhancer elements in the 9q31.2 locus. **a** The purple bar demonstrates the location of the rs10979182 LD block (*r*^2^ > 0.8) in the ~ 1 Mb gene desert between two gene clusters, shown by UCSC genes [40]. **b** The 90 SNPs in LD with rs10979182 are denoted by purple lines and H3K4me1, and H3K27ac ChIP-seq tracks from ENCODE are shown as peaks in GM12878 (red), H1-hESC (yellow), HSMM (green), HUVEC (light blue), K562 (dark blue), NHEK (purple) and NHLF (pink) cells [41]. The index SNP, rs10979182, is shown as a green arrow, and the SNP of interest, rs6477612, is shown as a black arrow. **c** Zoom-in of the putative enhancers 2–4 showing SNPs overlaying ENCODE regulatory marks: H3K4me1 and H3K27ac ChIP-seq (NHEK), DNase clusters and transcription factor ChIP clusters across 91 cell types as grey/black bars, where darkness indicates signal strength. For ChIP clusters, green lines indicate the highest scoring site of a FactorBook-identified canonical motif for the corresponding factor. The figure was made with the UCSC Genome Browser, GRCh37/hg19 [42]

First, we characterised the psoriasis-associated SNPs in 9q31.2 by mining publicly available epigenetic datasets and tools. There are ninety variants in tight LD (*r*^2^ > 0.8) with the lead GWAS SNP rs10979182 (1KG Phase 3 European) (Fig. 4a); several of which intersect modified histone marks (H3K4me1 and H3K27ac) in several cell types from ENCODE, corresponding with four putative enhancer elements overlapping H3K4me1 and H3K27ac occupancy (Fig. 4b). In primary human keratinocyte (NHEK) cells, enhancer histone marks were most prominent in enhancers 2–4 (Fig. 4c). The SNPs also overlap DNase hypersensitivity sites and transcription factor binding sites (Fig. 4c) that correspond with enhancer elements in NHEK according to ChromHMM [43].

No eQTLs were identified in the set according to Haploreg v4.1. RegulomeDB identified rs6477612, situated within the third putative enhancer, as the SNP with the highest putative regulatory potential with a score of 2a. rs6477612 is in tight LD (*r*^2^ = 0.92, 1KG EUR) with rs10979182 and was located within the HindIII fragment found to interact with *KLF4* in HaCaT cells in our CHi-C data (chr9:110810592-110816598; hg19), making it a prioritised SNP of interest.

### HiChIP data suggested that the interactions between KLF4 and psoriasis SNPs are active in HaCaT cells, but not in My-La cells

**Fig. 5:**
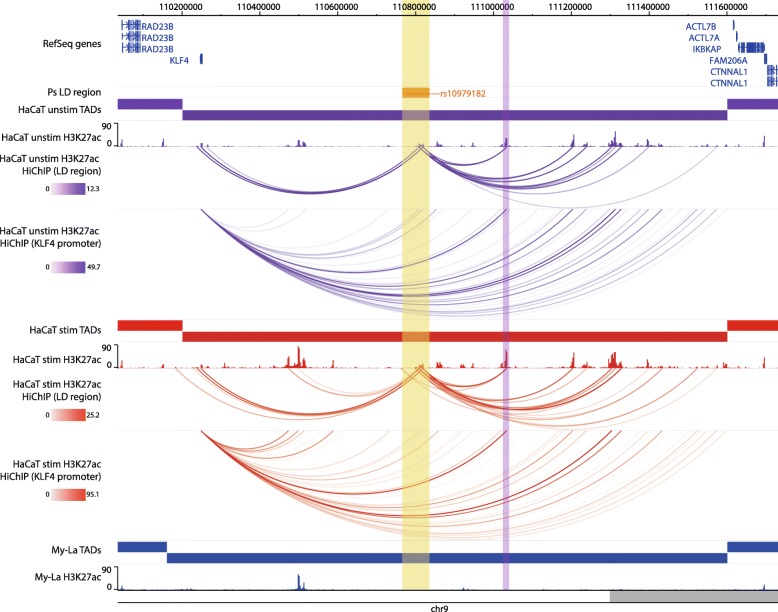
HiChIP (H3K27ac) interactions with the *KLF4* promoter in the 9q31.2 locus. The tracks include psoriasis LD block as defined by SNPs in *r*^2^ > 0.8 with rs10979182, RefSeq genes, TADs (shown as bars), H3K27ac occupancy (shown as peaks) and significant HiChIP interactions (shown as arcs) in three conditions: unstimulated HaCaT cells (purple), HaCaT cells stimulated with IFN-γ (red) and My-La cells (blue). The HiChIP interactions were restricted to those originating either from the psoriasis SNPS or the *KLF4* promoter. The yellow highlighted region indicates the psoriasis LD block at rs10979182. The purple highlighted region indicates the previously described *KLF4*-interacting region in the breast cancer study [17]. The scales in the HiChIP interactions represent the FitHiChIP score. The figure was made with the WashU Epigenome Browser, GRCh37/hg19 [31]

As a complementary approach to CHi-C, we used the recently developed HiChIP method to identify H3K27ac-mediated interactions in our cell lines. In HaCaT cells, there was a H3K27ac peak at the *KLF4* promoter that interacted with several regions across the gene desert including psoriasis-associated enhancers 3 and 4 and at the previously published interacting region from the breast cancer study in both unstimulated and stimulated HaCaT cells (Fig. 5) [17]. The H3K27ac peaks at the psoriasis SNPs also interacted with several other putative enhancers within the gene desert, but did not interact with other gene targets, mirroring the CHi-C architecture (Fig. 5). In contrast, there was a lack of H3K27ac peaks in My-La cells in 9q31.2 and, correspondingly, no significant HiChIP interactions. This lack of H3K27ac occupancy indicates a differential activation state in this region between HaCaT and My-La cells.

We observed an increase in the number and strength of H3K27ac peaks in the 9q31.2 TAD (chr9:110202281-111602280) in stimulated HaCaT cells compared with unstimulated HaCaT cells (Fig. 5). The number of peaks increased from 60 to 77, and there was a significant increase in the median peak signal from ~ 5.3 to ~ 9.5 in shared peaks (*P* < 0.0001, Wilcoxon matched-pairs signed-rank test). This also corresponded with an over 5-fold upregulation of gene expression upon IFN-γ stimulation in HaCaT cells (FC = 5.78; adj. *P* = 4.26 × 10^−8^). Combined, this suggests that inflammatory stimulation of HaCaT cells causes upregulation of *KLF4* that is mediated, or at least accompanied, by increased enhancer activity in 9q31.2.

### 3C-qPCR supplemented HiChIP/CHi-C findings in the 9q31.2 locus

We used 3C-qPCR in an effort to confirm the interaction between the psoriasis-associated putative enhancer 3 (rs6477612) and *KLF4* and to further prioritise regulatory SNPs. Our 3C experiment utilised both the enhancer and the *KLF4* gene as focus anchors, in both HaCaT and My-La cell lines. The enhancer-focused 3C experiment identified interaction peaks with regions approximately 2.5 kb and 8.7 kb downstream of *KLF4* in My-La and with the downstream 8.7-kb region alone in HaCaT (Additional file 8, Fig. S5).

The *KLF4*-focused 3C experiment showed that *KLF4* significantly interacted with several intergenic psoriasis-associated fragments, including the fragment containing the third putative enhancer (rs6477612), in HaCaT cells, but not in My-La cells (Additional file 9, Fig. S6). This corroborates the CHi-C data, which showed a more robust interaction between the enhancer and the *KLF4* gene in HaCaT cells (Fig. 1a). A positive control interaction linking a distal breast cancer-associated locus with *KLF4* [17, 18] demonstrated the strongest interaction with the *KLF4* promoter region in both cell types (Additional file 9, Fig. S6).

Taken together, the 3C results confirm a close spatial proximity between the psoriasis-associated SNPs and *KLF4* in 9q31.2. However, there is no clear peak of interaction among the LD block that would implicate some SNPs over others. In addition, stronger interactions were seen between *KLF4* and regions further upstream in the gene desert, which correlates with previous CHi-C findings in breast cancer cells [17] and Hi-C findings in NHEK cells [14] (illustrated in Additional file 10, Fig. S7).

### ChIP-qPCR confirmed the presence of regulatory histone modifications in 9q31.2 in HaCaT cells

**Fig. 6:**
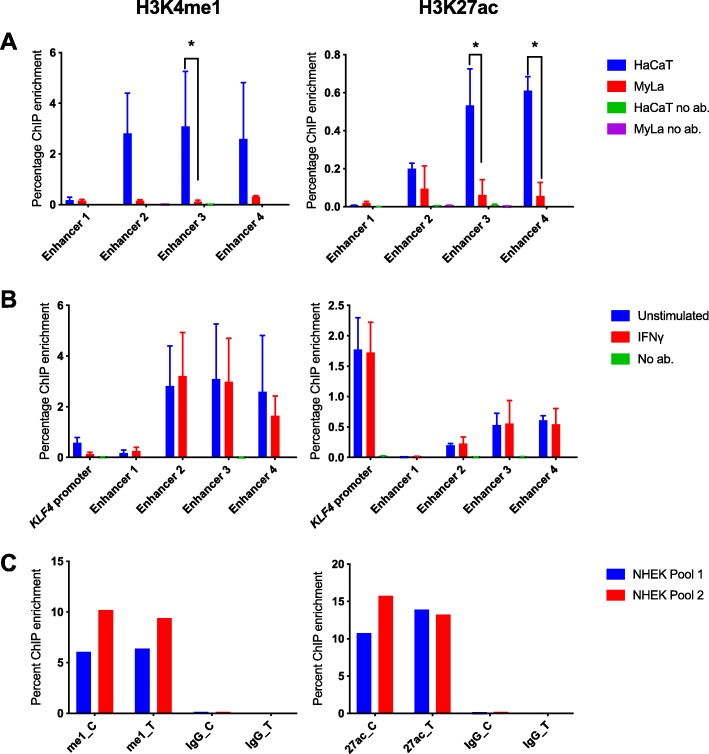
ChIP-qPCR for modified histone marks H3K4me1 and H3K27ac in 9q31.2. **a** Enhancer peaks defined by H3K27ac binding in ENCODE NHEK data were targeted in HaCaT cells (blue columns) and My-La cells (red columns). **b** Enhancer peaks were targeted in unstimulated (blue) and stimulated (red) HaCaT cells. The graphs show the mean ChIP enrichment of triplicate ChIP libraries ± SD, and samples with no antibody are consistently included for comparison, although they are often too low to be visible. To identify differential ChIP enrichment, 2-way ANOVA tests were performed in GraphPad prism using Sidak’s multiple comparisons test. Asterisks denote adjusted *P* < 0.05. **c** Allele-specific ChIP-qPCR for H3K27ac and H3K4me1 at rs6477612 in NHEK cells. Chromatin from two separate pools of NHEK cells, each comprising cells from three individual donors, was immunoprecipitated with H3K27ac (27 ac), H3K4me1 (me1) or non-specific IgG antibody (IgG), and qPCR was conducted using a TaqMan genotyping assay for rs6477612 detecting C (risk) or T (protective) alleles. Percentage ChIP enrichment was calculated by comparing the signal for each allele in the immunoprecipitated DNA with the signal for each allele in the input DNA for each of the two samples

We performed ChIP-qPCR of the histone marks H3K4me1 and H3K27ac to confirm the cell type specificity of enhancer activity within the *KLF4*-interacting psoriasis loci. Primers were designed to target 150–200-bp regions encompassing predicted peaks of H3K27ac occupancy in the four putative enhancers identified from ENCODE data (NHEK). H3K4me1 and H3K27ac occupancy was detected at all tested loci in HaCaT and My-La cells (Fig. 6a). However, occupancy was significantly increased in HaCaT cells with an enrichment of both H3K4me1 and H3K27ac at enhancer 3 (H3K4me1 *P* = 0.0372, H3K27ac *P* < 0.0001) and H3K27ac at enhancer 4 (*P* < 0.0001) in HaCaT cells in comparison with My-La cells (Fig. 6a). Stimulation of HaCaT cells with IFN-γ had little effect on the occupancy of H3K4me1 or H3K27ac at the regions tested within the enhancers, or at a region tested at the *KLF4* promoter (Fig. 6b).

To determine the potential effects of the risk or protective allele of rs6477612, a SNP of interest within enhancer 3, we performed allele-specific ChIP at rs6477612 for H3K4me1 and H3K27ac in two pools of NHEK cells. However, there was no discernible difference in H3K4me1 or H3K27ac occupancy at the risk (C) or protective (T) allele of rs6477612 (Fig. 6c).

In summary, by combining the HiChIP, CHi-C 3C and ChIP evidence, we could determine that the psoriasis-associated enhancer region interacts with *KLF4* in both My-La and HaCaT but is only active in HaCaT cells. Enhancer activity in 9q31.2 is increased after IFN-γ stimulation, correlating with an increase in *KLF4* gene expression, although we were unable to detect increases in H3K27ac occupancy at the tested psoriasis-associated enhancer regions.

### CRISPR activation suggested that the psoriasis-associated enhancer elements regulate KLF4 expression in 9q31.2

**Fig. 7:**
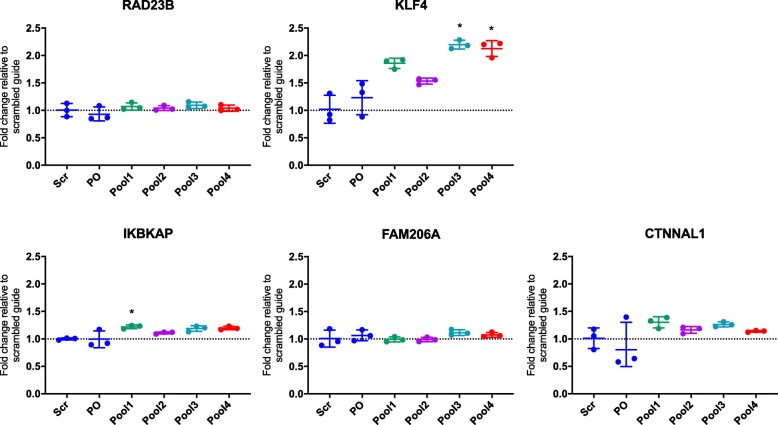
qPCR results for genes within the 9q31.2 locus in HaCaT cells expressing dCas9-P300. HaCaT cells expressing dCas9-P300 were transduced with pools of plasmids containing sgRNA targeting psoriasis SNPs (pools 1–4), a scrambled sgRNA (Scr) or the same plasmid without a specific guide cloned in (plasmid only (PO)). TaqMan qPCR results are shown for *RAD23B*, *KLF4*, *IKBKAP*, *FAM206A* and *CTNNAL1*. Housekeeping genes used were *TBP* and *YWHAZ*. For statistical analysis, a Kruskal-Wallis test was performed comparing the fold changes between cells with the scrambled guide or the sgRNA pools, using Dunn’s multiple comparisons test to identify significant differences. Asterisks denote *P* < 0.05. Graphs show the mean fold change in comparison with the scrambled guide, ± SD of biological triplicate cell lines

We employed CRISPRa in 9q31.2 to determine whether activating the psoriasis-associated enhancers could impact on gene expression (*KLF4* or other, more distal genes), implicating a functional role for the long-range interactions. Pools of single-guide RNA (sgRNA) targeting SNPs within the four psoriasis-associated enhancers were introduced into HaCaT cells stably expressing the CRISPR activator dCas9-P300 (see Additional file 11, Fig. S8 for overview of sgRNA locations). All four pools of sgRNA increased the *KLF4* expression in comparison with the control, scrambled sgRNA; this increase was statistically significant (after multiple testing correction) at enhancer 3 (*P* = 0.0143) and enhancer 4 (*P* = 0.0183) (Fig. 7). Pool 3, targeting enhancer 3 containing rs6477612, had the greatest impact with a 2.2-fold increase in the *KLF4* expression. To a lesser extent, IkappaB kinase complex-associated protein (*IKBKAP*) to the telomeric end of the gene desert was also subtly but significantly upregulated by approximately 1.2-fold in cell lines containing sgRNA pool 1 in comparison with the scrambled sgRNA (*P* = 0.0372). We found that *FAM206A* and *CTNNAL1* were not significantly affected by CRISPRa (Fig. 7). The remaining two genes, *ACTL7A* and *ACTL7B*, were not detectable in any HaCaT cell line, transduced or otherwise (for *ACTL7A*, all Ct values ≥ 33.4 and for *ACTL7B*, all Ct values ≥ 34.1).

To determine the transcriptome-wide effects of activating the psoriasis-associated enhancers in 9q31.2, RNA-seq was performed on the HaCaT dCas9-P300 cells expressing the sgRNA in pool 3 (putative enhancer 3) and compared with cells expressing the scrambled sgRNA (Additional file 12 Table S13). In line with the qPCR experiment, RNA-seq revealed an approximately 3-fold increased expression of *KLF4* in the pool 3 cells (FC = 2.92; adj. *P* = 0.0546) and an approximately 1.2-fold increase in *IKBKAP*, *CTNNAL1* and *FAM206A* expression, but these were not significant (IKBKAP FC = 1.26, adj. *P* = 0.7331; *CTNNAL1* FC = 1.23, adj. *P* = 0.68; *FAM206A* FC = 1.24, adj. *P* = 0.82). Therefore, *KLF4* is the only candidate in this locus with convincing evidence for *cis*-regulation by the targeted enhancers.

The RNA-seq analysis showed that there were an additional 236 differentially expressed genes in the CRISPRa experiment (adjusted *P* ≤ 0.10), 128 upregulated and 108 downregulated (Additional file 12 Table S13). Importantly, CRISPRa of the psoriasis-implicated enhancer in this keratinocyte cell line not only resulted in an increase in *KLF4* expression, but a differential expression of 3 keratin genes (keratin 4, 13 and 15), confirming the importance of this enhancer and the *KLF4* gene in skin cell function. Keratin 4 was the most differentially expressed gene in our data with keratin 15 being the 6th most differentially expressed. Previous studies also demonstrated a differential impact of *KLF4* on keratin gene regulation [44–46].

Confirming the importance of *KLF4* in skin cells and validating previous findings of differential gene expression with *KLF4* stimulation, differential genes also included *EREG* (an epidermal growth factor), *MMP13* (extracellular matrix protein gene) and *CLDN8* (claudin 8; important in epithelium tight junctions). We also demonstrated a ~ 10-fold reduction in the expression of *ALPG*, from a family of alkaline phosphatases showing the largest fold change in a previous *KLF4* over-expression study [44] (Additional file 12 Table S13). The upregulated genes were enriched in several biological pathways according to the GAGE pathway analysis, of which, the most significant related to RNA processing (Additional file 12, Table S14). According to the STRING database, differential genes were enriched in a number of relevant pathways including apoptosis and response to cell stress, emphasising the role that *KLF4* plays in cell cycle regulation and supporting previous findings demonstrating that over-expressing *KLF4* leads to G1/S cell cycle arrest [47] (Additional file 12, Table S15).

To confirm if the differential genes in the CRISPRa experiment were regulated by KLF4, we used a recently published tool that predicts and ranks transcriptional regulators of gene sets, Lisa [48]. The tool predicted that indeed the gene set was significantly regulated by KLF4 in keratinocytes, breast epithelium and skin fibroblasts. However, KLF4 was ranked number 104 of all the transcriptional regulators (Additional file 12, Table S16). We speculate that the CRISPRa experiment may yield many differentially expressed genes that are not directly regulated by KLF4, but rather are involved in the further downstream cascade of altered gene regulation.

Taken together, the chromatin interaction data coupled with the CRISPRa experiment allow for prioritisation of likely causal variants in 9q31.2 (Additional file 12 Table S17). From the 90 variants in LD with the index SNP, rs10979182, four variants interacted with the likely causal gene *KLF4* in both the CHi-C and HiChIP data and overlapped H3K27ac peaks (HaCaT cells): rs60082362, rs55975335, rs6477612 and rs6477613. These variants are all located in the CRISPRa pool 3, which marginally had the greatest impact on the *KLF4* expression.

## Discussion

Genetic predisposition is the largest known risk factor for psoriasis. The GWAS era has provided a wealth of genetic loci associated with psoriasis, and yet, if we are to exploit these data fully, for clinical benefit with new and more effective therapies, there is a requirement for a better understanding of their biological significance. Recent functional studies have used sophisticated post-GWAS technologies to assign gene targets, cell types and functional mechanisms in the loci associated with other, related complex conditions [20, 49–51]. Here, we have combined these technologies for the first time focused on investigation into the functional genomics of psoriasis.

Our study provides findings that are complementary to previously published data. As an example, our data in cell lines demonstrates a long-range interaction between psoriasis-associated SNPs and *PTGER4* in the intergenic psoriasis risk locus at 5p13.3, similar to published, promoter CHi-C data in several other primary cell types, including psoriasis-relevant cells such as macrophages, monocytes, CD4+ T cells, CD8+ T cells and neutrophils [13]. Recently, HiChIP again demonstrated how this locus forms functional enhancer interactions with *PTGER4* [22]. We also demonstrate how chromatin conformation data can provide evidence for suspected causal gene targets or provide support for regions that show an eQTL to a putative causal gene.

In the intergenic 9q31.2 psoriasis risk locus, we implicate *KLF4* as the likely causal psoriasis risk gene in this region by its functional interaction with the psoriasis GWAS variants. By incorporating RNA-seq analysis, we demonstrate that activating the disease implicated enhancers also activates downstream pathways of *KLF4*, showing how perturbation of a regulatory sequence and modest effect on transcription factor expression can have a profound effect on downstream gene targets in disease. KLF4 protein contains both an activation and repression domain and is known to either upregulate or downregulate pathways in a tissue- and context-dependent manner; therefore, its role in disease is likely to be complex [52], but it has already been shown by others to be dysregulated in psoriatic plaques [53]. Whilst our study was limited in its ability to identify a single causal variant, our findings did prioritise four variants from 90 in LD with the lead SNP thereby providing a solid basis for further follow-up using novel technologies such as fine-tuned CRISPR experiments.

## Conclusions

In conclusion, we provide evidence for putative gene targets in psoriasis risk loci, supporting assigned candidates and in some regions suggesting novel candidates. We also focus on a specific risk locus, and by moving from associated variants to gene, cell type mechanism and pathway, we demonstrate how *KLF4* is a likely gene target of the GWAS association in 9q31.2. This investigative pathway is applicable to all GWAS studies and helps make the next pivotal step towards patient benefit and clinical translation.

## Methods

### Cell culture

HaCaT keratinocyte cells were obtained from Addexbio (T0020001); these are in vitro spontaneously transformed keratinocytes from histologically normal skin. Cells were cultured in high-glucose Dulbecco’s modified Eagle’s medium (DMEM) supplemented with 10% foetal bovine serum (FBS) and penicillin-streptomycin (Thermo Fisher Scientific, final concentration 100 U penicillin, 0.1 mg streptomycin/mL). For HaCaT stimulation experiments, the media were supplemented with 100 ng/mL recombinant human IFN-γ (285-IF-100; R&D Systems) and cells incubated for 8 h prior to harvest.

Pools of adult normal human epidermal keratinocytes (NHEK) were obtained from PromoCell (C-12006) and cultured in Keratinocyte Growth Medium 2 (PromoCell) supplemented with 0.06 mM CaCl_2_.

My-La CD8+ cells were obtained from Sigma-Aldrich (95051033). These cells are cancerous human T lymphocytes derived from a patient with mycosis fungoides. Cells were cultured in Roswell Park Memorial Institute (RPMI) 1640 medium supplemented with 10% AB human serum (Sigma Aldrich), 100 U/mL recombinant human IL-2 (Sigma-Aldrich) and penicillin-streptomycin (final concentration 100 U penicillin, 0.1 mg streptomycin/mL).

Lenti-X 293T cells were obtained from Takara Biosciences (632180). These cells are a sub-clone of the human embryonic kidney (HEK) cell line and are optimised for viral protein production. 293T cells were cultured in DMEM high glucose supplemented with 10% FBS and penicillin streptomycin (final concentration 100 U penicillin, 0.1 mg streptomycin/mL).

### Cell crosslinking for chromatin-based experiments

HaCaT and NHEK cells were crosslinked for 10 min in 1% formaldehyde, and the reaction was quenched with 0.135 M glycine. The crosslinked cells were pelleted and washed in PBS and the supernatant removed. Cells were snap frozen on dry ice and stored at − 80 °C. My-La cells were crosslinked for 10 min in 1% (ChIP, HiChIP) or 2% (Hi-C, 3C) formaldehyde. The reaction was quenched with 0.135 M glycine, the supernatant removed and the cells snap frozen on dry ice and stored at − 80 °C.

### Capture Hi-C

For CHi-C, RNA baits were designed to target all known non-MHC psoriasis risk loci, defined by one or more independent SNPs associated with psoriasis in GWAS (Additional file 1, Table S1). The total number of SNPs included was 107 (59 associated with Europeans, 42 with Chinese, and 6 associated with both European and Chinese cohorts) corresponding with 68 loci. The baits were selected to target HindIII fragments that overlapped with the linkage disequilibrium (LD) block in each locus, defined by SNPs in *r*^2^ > 0.8 with the lead SNP (1000 Genomes Phase 3 release, European). Due to sequence restraints, baits could not be designed for the 1p36.11 (rs7552167, rs4649203) [30, 54] and 1q31.1 (rs10789285) [32] loci; therefore, in total, there were 104 SNPs corresponding with 66 psoriasis risk loci in the final capture library (907 HindIII fragments).

The psoriasis baits were designed as part of a capture library targeting multiple GWAS loci across several immune-mediated diseases: juvenile idiopathic arthritis, asthma, psoriatic arthritis, rheumatoid arthritis and systemic sclerosis. The majority of these baits were included in our previous region CHi-C experiment [20]. This initial capture library was applied to My-La Hi-C libraries, whereas a slightly updated capture library, which included some additional baits, was applied to HaCaT Hi-C libraries. The two different baitmaps included the same psoriasis regions, and results from non-psoriasis loci are not described in the present study. In addition, a control locus, which represents a well-characterised region of long-range interactions, *HBA*, was also included (174.57 kb genomic, 26 restriction fragments, 6.71 kb/restriction fragment). Each 120-bp bait was targeted to within 400 bp of a HindIII fragment end, comprised 25–65% GC content and contained fewer than three unknown bases. The baits were synthesised by Agilent Technologies.

CHi-C libraries were generated in biological duplicate for My-La and HaCaT (unstimulated or stimulated) cells according to previously described protocols [17, 20]. Fifty million crosslinked cells were lysed and the chromatin digested with HindIII at 37 °C overnight. Restriction cut sites were filled in using dCTP, dGTP, dTTP and biotin-14-dATP (Life Technologies), then in-nucleus ligation was carried out at 16 °C for 4–6 h. Crosslinks were removed by proteinase-K overnight at 65 °C, and RNA was digested using RNaseA for 60 min at 37 °C. The DNA was purified by sequential phenol and phenol-chloroform extractions and ethanol-precipitated at − 20 °C overnight, followed by two further phenol-chloroform extractions and a second overnight precipitation.

A 40-μg aliquot of DNA was taken forward for further processing following QC steps. T4 DNA polymerase was used to remove biotin-14-dATP from non-ligated ends then the DNA purified by phenol-chloroform extraction and ethanol precipitation overnight. The DNA was sheared using a Covaris S220 sonicator, and end-repair was performed using T4 DNA polymerase, T4 DNA polynucleotide kinase and DNA polymerase I, large (Klenow) fragment. The sample was purified using Qiagen MinElute Kit, with a modified protocol described by [55]. Klenow (exo-) was used to adenylate DNA fragment ends, and a double-sided SPRI bead size selection was used to obtain fragments of approximately 200–600 bp. Dynabeads MyOne Streptavidin C1 beads (Life Technologies) were used to pull down biotinylated fragments, which were then ligated to annealed Illumina sequencing adapters. PCR was performed using Phusion HF (NEB) and TruPE PCR primers (Illumina), then the amplified DNA was cleaned twice using 1.8× volume of SPRI beads.

Amplified DNA up to 750 ng was concentrated using a vacuum concentrator and bound to the capture baits in a single hybridisation reaction using SureSelectXT reagents and protocol by Agilent Technologies. The biotinylated baits were captured using Dynabeads MyOne Streptavidin T1 beads (Life Technologies). Following washes, the libraries were amplified on the beads using Phusion HF and barcoded TruPE primers then the amplified DNA cleaned twice using 1.8× volume of SPRI beads. The quality and quantity of the Capture Hi-C libraries were tested by Bioanalyzer and KAPA qPCR (Kapa Biosystems). Capture Hi-C libraries were analysed by 75-bp paired-end next-generation sequencing on an Illumina NextSeq500 at the Genomic Technologies Core Facility at the University of Manchester (My-La) or HiSeq 4000 at Edinburgh Genomics at the University of Edinburgh (HaCaT).

CHi-C sequence data were processed through the Hi-C User Pipeline (HiCUP) v0.5.8 [56]. For each cell type, the two biological replicates were simultaneously run through CHiCAGO v1.1.8 [57] in R v3.3.0, and significant interactions were called with a score threshold of 5. To down-sample My-La libraries in order to more closely mirror the number of HaCaT on-target di-tags, we used DownsampleSam from Picard v2.9.2 [58] with the option *P* = 0.3 (replicate 1) and *P* = 0.4 (replicate 2), followed by HiCUP and CHiCAGO pipelines as above. Reproducibility was assessed firstly by observing the number of shared interactions between replicates. We generated Venn diagrams displaying the shared interactions (CHiCAGO score ≥ 5) between each replicate called individually, or both replicates combined in the CHiCAGO function. We used HiCRep [25] to assess the correlation between samples by firstly filtering for reads with a captured bait at one or both ends, using the shared baits across samples, and then creating 10-kb raw matrices on chromosome 1 using HOMER [59]. Matrices were submitted to HiCrep in all pairwise combinations with the following parameters: resolution = 10 kb, smoothing parameter (*h*) = 3 and maximum distance = 5 Mb.

To detect enrichment of features in the CHi-C interactions, we first obtained narrowPeak bed files of H3K4me3, H3K27ac and CTCF as follows: H3K27ac in NHEK (ENCODE; ENCFF943CBQ), H3K4me3 in NHEK (ENCODE; ENCFF027FJC), H3K27ac in primary CD8+ naive T cells (Roadmap Epigenomics; E047), H3K4me3 in primary CD8+ naive T cells (Roadmap Epigenomics; E047) and CTCF in NHEK (ENCODE; ENCFF226MQR). We obtained transcription start sites (Ensembl 99) for all genes expressed in the corresponding cell type in our RNA-seq data using Ensembl BioMart. The “peakEnrichment4Features” function in CHiCAGO was used to detect enrichment of each feature in the CHi-C data.

To determine the differences in the interaction distances, we restricted the data to psoriasis loci and obtained all distances in *cis* for each cell line. We then performed a Kruskal-Wallis test using Dunn’s multiple comparisons to determine cell-specific differences. BEDTools v2.17.0 [60] was used to detect interactions between psoriasis-associated fragments and gene promoters, defined by fragments covering regions within 500 bp of transcription start sites (Ensembl release 75; GRCh37). To validate our CHi-C data, we overlapped the interactions with psoriasis GWAS SNPs colocalised with eQTLs [27]. Sixteen out of 26 of their reported proxies overlapped HindIII fragments in our CHi-C data; the lack of complete overlap mostly owed to our study prioritising index SNPs from more recent meta-analyses over older studies. In addition, we were not able to design baits for one of the reported regions at rs7552167, as described above. We then determined how many of the target genes were implicated by CHi-C interactions, as visualised on the WashU Epigenome Browser [31]. We collated a list of all psoriasis GWAS SNPs or proxies that fell within baited fragments interacting with expressed genes (*N* = 1776) and determined if they altered known transcription factor binding motifs using the tool SNP2TFBS [29].

### Hi-C

For each cell type, a single Hi-C library was generated by re-amplifying the pre-Capture Hi-C library bound to streptavidin beads, using Phusion HF and barcoded TruPE primers. The amplified DNA was cleaned twice using 1.8× volume of SPRI beads. The quality and quantity of the Hi-C libraries were tested by Bioanalyzer and KAPA qPCR. Hi-C libraries were analysed by next-generation sequencing. The My-La Hi-C library was sequenced on an Illumina HiSeq 2500 generating 100-bp paired ends at the Babraham Institute Sequencing Facility, Cambridge. The HaCaT Hi-C libraries were sequenced on an Illumina HiSeq 4000 generating 75-bp paired ends at Edinburgh Genomics at the University of Edinburgh. The sequence data was filtered, and the adapters were removed using fastp v0.19.4 [61]. The reads were then mapped to the GRCh38 genome with Hi-C Pro v2.11.0 [62], using default settings. The Hi-C interaction matrices were normalised within Hi-C Pro using iterative correction and eigenvector decomposition (ICE). Topologically associating domains (TADs) were then called in the TADtool software [63] using insulation score with the normalised Hi-C contact matrices, binned with 40 kb resolution. TADs were visualised alongside CHi-C interactions on the WashU Epigenome Browser [31].

### HiChIP

For each cell type, HiChIP libraries were generated according to the Chang Lab protocol [21] in biological duplicate. Ten million crosslinked cells were lysed and the chromatin digested using 375 U of MboI (NEB, R0147M) for 4 h at 37 °C. Fragment ends were filled in using dCTP, dGTP, dTTP and biotin-14 dATP (Life Technologies) and ligated at room temperature overnight. The nuclei were lysed and the chromatin sheared to lengths of approximately 200–700 bp using a Covaris S220. Immunoprecipitation was performed overnight at 4 °C using 20 μg of H3K27ac antibody (Abcam ab4729). The DNA was captured on a 1:1 mixture of protein A and G Dynabeads (Invitrogen 10001D and 10003D). After washes, the DNA was eluted with proteinase K at 65 °C overnight. The sample was cleaned using Zymo Clean and Concentrator Columns (Zymo D4013) and quantified using the Qubit DNA HS kit. Twenty to 35 ng of DNA was taken forward for biotin pulldown with streptavidin C-1 beads at room temperature for 30 min. The beads were suspended in TD buffer from the Nextera kit and transposed with Tn5 (Illumina) at 55 °C for exactly 10 min. The volume of Tn5 was dependent on DNA quantity and defined by the original HiChIP protocol [21]. After washes, the library was amplified off the beads using Phusion polymerase and Nextera indexing primers (Illumina). SPRI beads were used to select fragments approximately 300–700 bp in length. Quantification and quality control of the final HiChIP library was conducted using a Bioanalyzer and KAPA quantification kit (Kapa Biosystems). Libraries underwent next-generation sequencing on a HiSeq 2500 at the Babraham Institute Sequencing Facility, Cambridge, generating 100-bp paired ends.

Sequencing data for the HiChIP libraries was filtered, and the adapters were removed using fastp v0.19.4. The reads were then mapped to the GRCh38 genome with Hi-C Pro v2.11.0, using default settings. The two replicates were merged, and loops were identified using FitHiChIP [64] using the following settings: coverage normalisation, stringent background with merging enabled, peaks generated from HiChIP data using the included tool and 5-kb bin size. The interactions were filtered to those originating from the H3K27ac peak on the *KLF4* promoter, or the psoriasis SNPs, before being uploaded for visualisation on the WashU Epigenome Browser [31].

To compare H3K27ac signal in shared peaks between unstimulated and stimulated HaCaT cells in 9q31.2, genome-wide anchors were called in hichipper v0.7.3 [65] using the self-circle and dangling end reads for the first replicate, extended for 147 bp. These peaks were first combined to produce a merged peak set, and then the signal from the two conditions was intersected on the peaks using BEDTools map function and the mean signal for each peak was reported for each condition. The resulting values were imported in R and normalised using DESeq2 estimate size factors function [66]. The normalised counts for peaks within the 9q31.2 TAD (chr9:110202281-111602280) were compared between the two conditions using a Wilcoxon matched-pairs signed-rank test in GraphPad Prism.

### RNA-seq

3′ mRNA sequencing libraries were generated for cell lines using the QuantSeq 3′ mRNA-Seq Library Prep Kit FWD for Illumina (Lexogen). RNA-seq libraries were generated for unstimulated HaCaT cells (*N* = 4), stimulated HaCat cells (*N* = 3) and My-La cells (*N* = 1). Libraries were sequenced using single-end Illumina SBS technology. Reads were quality trimmed using Trimmomatic v0.38 [67] using a sliding window of 5 with a mean minimum quality of 20. Adapters and poly A/poly G tails were removed using Cutadapt v1.18 [68], and then UMIs were extracted from the 5′ of the reads using UMI-tools v0.5.5 [69]. Reads were then mapped using STAR v2.5.3a [70] on the GRCh38 genome with GENCODE annotation v29. Reads were de-duplicated using UMIs with UMI-tools and then counted using HTSeq v0.11.2 [71]. Count matrixes were analysed in R 3.5.1, and normalisation and differential expression analysis was conducted using DESeq2 v1.22.2. Differentially expressed genes were called with an adjusted *P* value of 0.10 (FDR 10%). Gene set enrichment pathway analysis was performed using GAGE v2.32.1 [72] using “normal” shrinked log fold changes from DESeq2. For the detection of expressed genes in the cell lines, we considered RNA-seq counts greater than 1 in at least one of the sequenced samples.

### Functional annotation in 9q31.2

SNPs in LD (*r*^2^ > 0.8) with the lead SNP rs10979182 were examined for their intersection with ENCODE data for histone marks, transcription factor binding sites and DNase hypersensitivity. RegulomeDB v1.1 [73] was used to rank the SNPs based on the likely regulatory function. The SNPs were also assessed using Haploreg v4.1 [74], which includes expression quantitative trait loci (eQTL) data from several studies including GTEx [75] and GEUVADIS [76].

### 3C-qPCR in 9q31.2

3C libraries were generated in biological triplicate as previously described [77]. Twenty to 30 million crosslinked cells were lysed, digested, ligated and purified as described in the first section of the Capture Hi-C protocol above, omitting the biotinylation step. Control libraries were constructed using bacterial artificial chromosome (BAC) clones as described by [77]. A set of 11 minimally overlapping BAC sequences was compiled to span the locus (chr9:110168556-111889073, hg19): RP11-795I4, CTD-2258 L2, RP11-762G1, RP11-358A7, RP11-779 J13, CTD-2517A7, RP11-454G15, RP11-585H18, CTD-2333H8, CTD-2649 N21 and RP11-316H9. BAC clone identity was confirmed by PCR to amplify selected regions at either end of the sequence. BAC DNA was combined in equimolar quantities to a total of 20 μg and digested with HindIII overnight at 37 °C. The DNA was purified with phenol-chloroform and precipitated in ethanol for several hours at − 20 °C. Ligation was performed at 16 °C overnight using T4 DNA ligase. Two further phenol-chloroform extractions were performed followed by a chloroform extraction, and the DNA was precipitated in ethanol for several hours at − 20 °C. 3C libraries and BAC control libraries were quantified using a Qubit dsDNA BR kit.

qPCR was carried out using SYBR Green or TaqMan technology to determine the interaction frequencies in the 9q31.2 locus. Unidirectional primers were designed using Primer3 (http://primer3.ut.ee/) [78] to complement the sequences approximately 50–100 bp from the target HindIII cut site (primers shown in Additional file 13 Table S18). For the TaqMan experiment, an additional TaqMan probe was designed to bind to a region between the anchor primer and the restriction cut site (probe and primers shown in Additional file 13 Table S19). For each anchor fragment, a primer was designed to target a short-range control region located 2–3 fragments further along the sequence. qPCRs were carried out in technical triplicate, and 10-fold dilutions of the BAC template (50–0.005 ng) were included alongside the 3C library templates for each tested interaction. For each primer pair, BAC curves were generated from Log_10_ of the library concentration against the average Ct value across PCR triplicates. The relative interaction frequencies were calculated in the following manner:

$$ \mathrm{Interaction}\ \mathrm{frequency}\ (F)={10}^{\left(\left(\mathrm{Ct}-i\right)/s\right)} $$

where Ct is the measured cycle threshold value of 3C library (mean of PCR triplicates), *i* is the *Y* intercept of BAC curve and *s* is the slope of BAC curve.

The interaction frequencies were then normalised to the short-range control as follows:

$$ \mathrm{Relative}\ \mathrm{interaction}\ \mathrm{frequency}\ (R)=\frac{F\left[\mathrm{short}-\mathrm{range}\ \mathrm{control}\right]}{F\left[\mathrm{test}\ \mathrm{interaction}\right]} $$

where *F* is the interaction frequency.

Significant interactions were detected using one-way ANOVA in GraphPad Prism 7 with Dunnett’s or Tukey’s test for multiple comparisons dependent on whether there was a single negative control region or each interaction was compared to all other interactions, respectively.

### ChIP-qPCR in 9q31.2

Chromatin immunoprecipitation (ChIP) libraries were generated as previously described [49]. Ten million crosslinked cells were lysed, and the chromatin was fragmented to optimal lengths of 200–400 bp using a Covaris S220. A volume of chromatin corresponding to approximately 1 million cells was immunoprecipitated with a rabbit polyclonal antibody for H3K4me1 (Abcam ab8895) or H3K27ac (ab4729) or a negative control rabbit IgG (Diagenode C15410206) with a mixture of Dynabeads A and G at 4 °C overnight. After washes, the DNA was eluted with proteinase K at 62 °C for 2 h and then 95 °C for 10 min. The DNA was purified using Purelink Quick PCR purification columns (Life Technologies). ChIP enrichment was measured at loci of interest by qPCR using SYBR Green or TaqMan technology. The data were normalised by calculating the percentage of total chromatin that was immunoprecipitated in comparison with an input sample. Negative controls included no-antibody and IgG-precipitated samples.

For SYBR experiments, primers were designed using Primer3 (http://primer3.ut.ee/) to target regions of 100–200 bp encompassing likely regulatory SNPs or enhancers in 9q31.2 (for primers, see Additional file 13, Table S20). For the allele-specific analysis at rs6477612, a TaqMan SNP genotyping probe was acquired that detected C (risk) or T (protective) alleles (Applied Biosystems, assay ID C__29343482_10). The difference in antibody binding to each allele was determined by the percentage of ChIP enrichment in comparison with the signal for each allele obtained from the input sample. ChIP-qPCR data were analysed in GraphPad Prism using two-way ANOVA. Experiments were performed in biological triplicate (HaCaT and My-La lines) or duplicate (NHEK cells).

### CRISPR activation in 9q31.2

CRISPR activation using the catalytically inactive Cas9 (dCas9)-P300 complex was performed in HaCaT cells to determine the role of the four putative enhancers in 9q31.2. Firstly, a HaCaT cell line stably expressing dCas9-P300 was generated using the CRISPRa plasmid pLV_dCas9-p300-P2A-PuroR plasmid (Addgene 83889) [79]. Briefly, plasmid DNA was combined with third-generation viral packaging components and polyethyenimine (PEI) in the ratio 1:6 (total DNA:PEI). The DNA:PEI complexes were added to Lenti-X 293T cells, which were then incubated for 72 h, after which the lentivirus-containing supernatant was harvested and filtered to remove cell debris. Lentiviral transduction of 300,000 HaCaT cells was carried out using 1 mL of the unconcentrated lentivirus and 8 μg/mL polybrene. The cells were grown for several days before selection with 1 μg/mL puromycin for 7 days, after which the HaCaT dCas9-P300 cell population was maintained with 0.5 μg/mL puromycin.

To select sgRNA in 9q31.2, SNPs in *r*^2^ > 0.8 with rs10979182 were prioritised by their overlap with enhancer elements, defined by active regulatory regions in NHEK according to ChromHMM [11]. sgRNA sequences were designed using the online CRISPOR tool [80] to target loci within 200 bp of the prioritised SNPs (mean = 85 bp). sgRNA were selected based on specificity score and proximity to the SNP. In total, there were 27 SNPs; two of these could not be targeted by sgRNA within 200 bp (rs7019552 and rs11355519), and another two SNPs, rs4979624 and rs7029094, were captured by a single sgRNA targeting the intervening region. In total, therefore, there were 24 sgRNA; these were grouped into four pools of 5–7 SNPs to target the four putative enhancers (Additional file 13, Table S21 and Additional file 11, Fig. S8).

For each of the sgRNA pools and controls (scrambled guide or empty plasmid), we generated three biological replicate cell lines as follows. The sgRNA sequences were cloned into the pLKO5.sgRNA.EFS.GFP plasmid (Addgene 57,822) [81], and equimolar plasmid pools were generated for each enhancer. The plasmid pools were then packaged using the same lentiviral method as the dCas9-P300 plasmid. The guide pools were introduced into the stable HaCaT dCas9-P300 cells using a second round of lentiviral transduction, and cells that had integrated the sgRNA plasmids were isolated by flow cytometry for GFP. RNA was extracted from the sorted cells using the RNeasy mini kit (Qiagen). qPCR was performed to assay gene expression using the TaqMan RNA-to-Ct 1-step kit (Thermo Fisher Scientific) using the following TaqMan assays for genes in the 9q31 locus: *RAD23B* (Hs00234102_m1), *KLF4* (Hs00358836_m1), *ACTL7A* (Hs00246418_s1), *ACTL7B* (Hs00246411_s1), *IKBKAP* (Hs00175353_m1), *FAM206A* (Hs00607423_m1) and *CTNNAL1* (Hs00972098_m1). Delta-delta Ct analysis was conducted against a control HaCaT dCas9-P300 cell line transduced with the sgRNA plasmid containing a previously published scrambled, non-targeting insert (Scramble2 [82];). Two housekeeping gene assays, *TBP* (Hs00427620_m1) and *YWHAZ* (Hs01122445_g1), were used for normalisation. For statistical analysis, we performed a Kruskal-Wallis test of the fold changes and used Dunn’s multiple comparisons test to determine the differences between cells with the scrambled guide or with the pools of sgRNA.

For the CRISPRa pool with the greatest impact on *KLF4* expression, RNA-seq and gene set enrichment analyses were performed as described above. In addition, differentially expressed genes were processed through the STRING database to identify potential protein-protein interaction networks [83]. Next, we used Lisa to search for transcriptional regulators of the differentially expressed gene set [48]. The genes were uploaded to the Lisa portal, and the combined model, based on CistromeDB TR ChIP-seq, was retrieved.

In addition to the above, the validity of the HaCaT dCas9-P300 system was first tested using sgRNA directed to the promoters of *IL1RN* or *SLC4A1*; these sgRNA have previously been shown to be effective in CRISPRa (IL1RN: sgRNA CR3 [84]; SLC4A1: Weissman Lab). The upregulation of these genes was detected by TaqMan qPCR for IL1RN (Hs00893626_m1) and SLC4A1 (Hs00978607_g1) (Additional file 14, Fig. S9).

## Supplementary information

**Additional file 1 **: **Table S1.** Psoriasis SNPs included in the capture design. **Table S2.** CHi-C library metrics.

**Additional file 2 **: **Figure S1.** Reproducibility between CHi-C replicates for HaCaT (unstimulated), HaCaT (stimulated with IFN-γ), and My-La (down-sampled). A) The number of shared cis-interactions (CHiCAGO score ≥ 5) between replicates are shown in a scaled Venn diagram for each cell type. Each CHi-C replicate was individually analysed by CHiCAGO, as well in the combined analysis where both replicates were submitted to CHiCAGO together. B) Correlation between CHi-C samples was assessed using HiCRep [25] for 10 kb interaction bins on chromosome 1. The heatmap shows the reported stratum-adjusted correlation coefficient (SCC) between samples.

**Additional file 3 **: **Figure S2.** Enrichment of features within other-ends of CHi-C interactions. The peak locations of H3K4me3, H3K27ac and CTCF in NHEK (ENCODE), H3K4me3 and H3K27ac in primary CD8+ naïve T cells (Roadmap Epigenomics) [85] and transcription start sites (Ensembl 99) of active genes (read counts > 0) according to the RNA-seq data in HaCaT and My-La cell lines in the present study were tested against other-ends of interactions with all targeted autoimmune loci using the peakEnrichment4Features function of the CHiCAGO package [57]. The graphs show the number of overlaps with the feature in the interaction data (yellow) versus the mean number of overlaps in 100 sampled interactions from the non-significant pool (blue). Error bars show the 95% confidence interval.

**Additional file 4 **: **Figure S3.** Frequency distributions of distances between psoriasis bait fragments and interacting fragments in the CHi-C experiment. The frequency of interactions is shown for 50 kb bins up to 3 Mb in HaCaT unstimulated (A), HaCaT stimulated (B) and My-La cells (C).

**Additional file 5 **: **Table S3.** Validation analysis of known eQTLs within the CHi-C data. **Tables S4-6.** CHi-C interactions between psoriasis loci and gene promoters with associated expression data. For each locus, the top interaction is shown between the psoriasis bait fragment and the gene promoter fragment in HaCaT unstimulated (S4), stimulated (S5) and My-La (S6).

**Additional file 6 **: **Figure S4.** Frequency distributions of the number of interactions with promoter fragments per psoriasis-associated bait fragment in the CHi-C experiment. To determine the frequency distribution of psoriasis bait-promoter interactions, the data was firstly restricted to interactions between psoriasis-associated bait fragments and promoter fragments (“Promoter Interactions”). Next, the number of promoter fragments per bait fragment was counted. Of those promoter fragments, the number of corresponding gene promoters was determined. This was necessary because some gene promoters share the same fragment, and some gene promoters are found in more than one fragment. The number of interacting promoter fragments per bait fragment in Promoter Interactions are shown for HaCaT unstimulated (A), HaCaT stimulated (C) and My-La (E). The number of corresponding gene promoters are shown for HaCaT unstimulated (B), HaCaT stimulated (D) and My-La (F). The interaction frequencies are shown in bins of 1.

**Additional file 7 **: **Table S7.** RNA-seq data: all normalised counts across the three cell lines. **Table S8.** Lists of expressed genes intersecting psoriasis bait fragments. **Table S9**. Enrichment of TFBSs among psoriasis GWAS SNPs interacting with promoters of active genes, using SNP2TFBS tool. **Table S10**. Differentially expressed genes between unstimulated and stimulated (IFNg) HaCaT cells. **Table S11**. GO term enrichments for DE genes in stimulation experiment. **Table S12**. DE genes interacting with psoriasis baits in unstimulated and stimulated HaCaT cells.

**Additional file 8 **: **Figure S5.** 3C-qPCR results in the 9q31.2 locus anchored at the HindIII fragment containing the third psoriasis-associated putative enhancer (rs6477612). qPCR was carried out on HaCaT and My-La 3C libraries using SYBR® Green as the reporter. The anchor fragment at the third psoriasis-associated enhancer is at distance 0 kb. Test fragments were selected in and around *KLF4*, two points in the gene desert and at fragments containing gene promoters for *IKBKAP*, *FAM206A* and *CTNNAL1*. Interactions were normalised to a short range control. Asterisks denote fragments that had a significantly higher relative interaction frequency than one or more of the other tested fragments, after multiple testing (one-way ANOVA, adjusted *P*-value < 0.05). Bars show mean + SD of triplicate 3C libraries. Abbreviations: Cent, centromeric; Int, intergenic.

**Additional file 9 **: **Figure S6.** 3C-qPCR results in the 9q31.2 locus from the HindIII fragment containing the *KLF4* gene and promoter. qPCR was carried out on HaCaT and My-La 3C libraries using TaqMan® as the reporter. The anchor fragment (distance 0) contained the entire *KLF4* gene and promoter. An intergenic fragment located approximately 200 kb from the anchor fragment was utilised as a negative control region. Eleven test fragments were selected at regular intervals across the psoriasis association. The positive controls in the Dryden BrCa region were included. Asterisks denote fragments that had a significantly higher relative interaction frequency than the NCR (one-way ANOVA, adjusted P-value < 0.05). Bars show mean + SD of triplicate 3C libraries. Abbreviations: Int, intergenic; NCR, negative control region; BrCa, breast cancer.

**Additional file 10 **: **Figure S7.** Previously reported HiC interaction data in NHEK cells in the 9q31.2 locus [14]. Interactions are indicated between *KLF4* and the gene desert, including the psoriasis SNPs and the breast cancer region shown in a previous CHi-C experiment [17]. Image created using the YUE lab 3D Genome Browser.

**Additional file 11 **: **Figure S8.** sgRNA pools targeting the four putative enhancers in the psoriasis susceptibility locus at 9q31.2. a) location on chromosome 9; b) sgRNA locations; c) SNPs in LD with rs10979182 overlapping putative enhancers; d) ChromHMM segments in NHEK where red, yellow and green indicate “active TSS”, “enhancers” and “transcription” respectively; e) H3K4me1 (ENCODE); f) H3K27ac (ENCODE); g) DNase clusters (ENCODE); h) transcription factor ChIP (ENCODE).

**Additional file 12 **: **Table S13.** Differentially expressed genes between HaCaT cells containing dCas9 P300 with scrambled guide or 9q31.2 Pool 3 guide in CRISPRa experiment. **Table S14.** GO term enrichments for DE genes in CRISPRa experiment. **Table S15.** STRING results for DE genes in CRISPRa experiments. **Table S16**. TF binding sites in DE genes (LISA result). **Table S17**. Annotated variants in LD with rs10979182 in 9q31.2.

**Additional file 13 **: **Table S18.** Primers used in the first 9q31.2 3C (SYBR) assay. **Table S19.** Primers used in the second 9q31.2 3C (TaqMan) assay. **Table S20**. Primers used in ChIP experiments. **Table S21**. sgRNA used in the CRISPRa experiment.

**Additional file 14 **: **Figure S9.** Effect of sgRNA targeting *IL1RN* and *SLC4A1* promoters in HaCaT dCas9 P300 cells. HaCaT cells expressing dCas9 P300 were transduced with sgRNA plasmids containing previously-published sgRNA for the *IL1RN* promoter or the *SLC4A1* promoter, in biological triplicate. Control cell lines were generated by transducing HaCaT dCas9 P300 cells with a sgRNA plasmid containing a scrambled guide (Scr) or no guide insert (Plasmid only; PO). qPCR was carried out using TaqMan assays for *IL1RN* or *SLC4A1*. Housekeeping genes used were *TBP* and *YWHAZ*. Graphs show fold change of gene expression relative to the cells containing the scrambled sgRNA. One-way ANOVA was carried out in GraphPad Prism: in both cases, the cells containing the targeting sgRNA had significantly higher gene expression than the scrambled control (*P*=0.002 for *IL1RN*; *P*=0.0001 for *SLC4A1*). Asterisks denote *P* < 0.05. Graphs show the mean fold-change in comparison with scrambled guide, +- SEM of triplicate cell lines.

## Data Availability

The sequence datasets generated and analysed during the current study are available in the GEO repository under accession number GSE137906.
