# Supplementary figures and images for: Mapping DNA interaction landscapes in psoriasis susceptibility loci highlights KLF4 as a target gene in 9q31
Source: BMC Biol. 2020 May 4;18:47. doi: 10.1186/s12915-020-00779-3 (PMC7199343; doi:10.1186/s12915-020-00779-3)

A

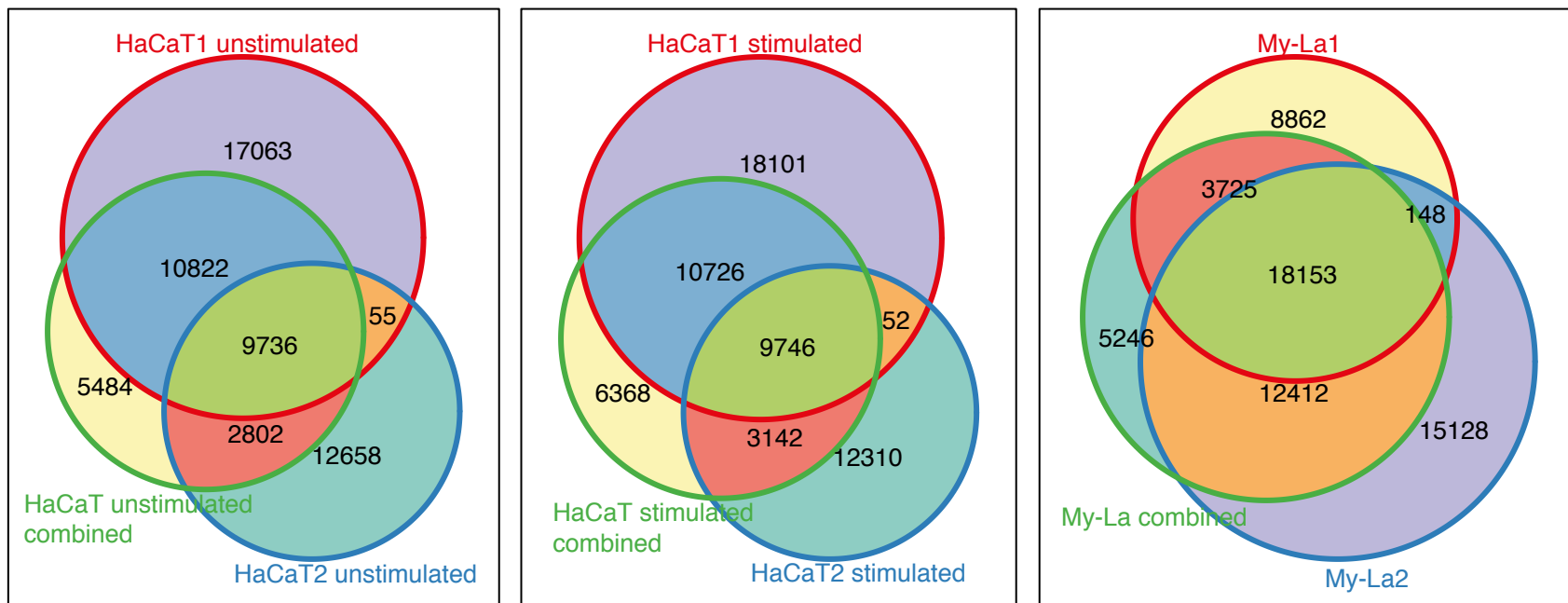

B

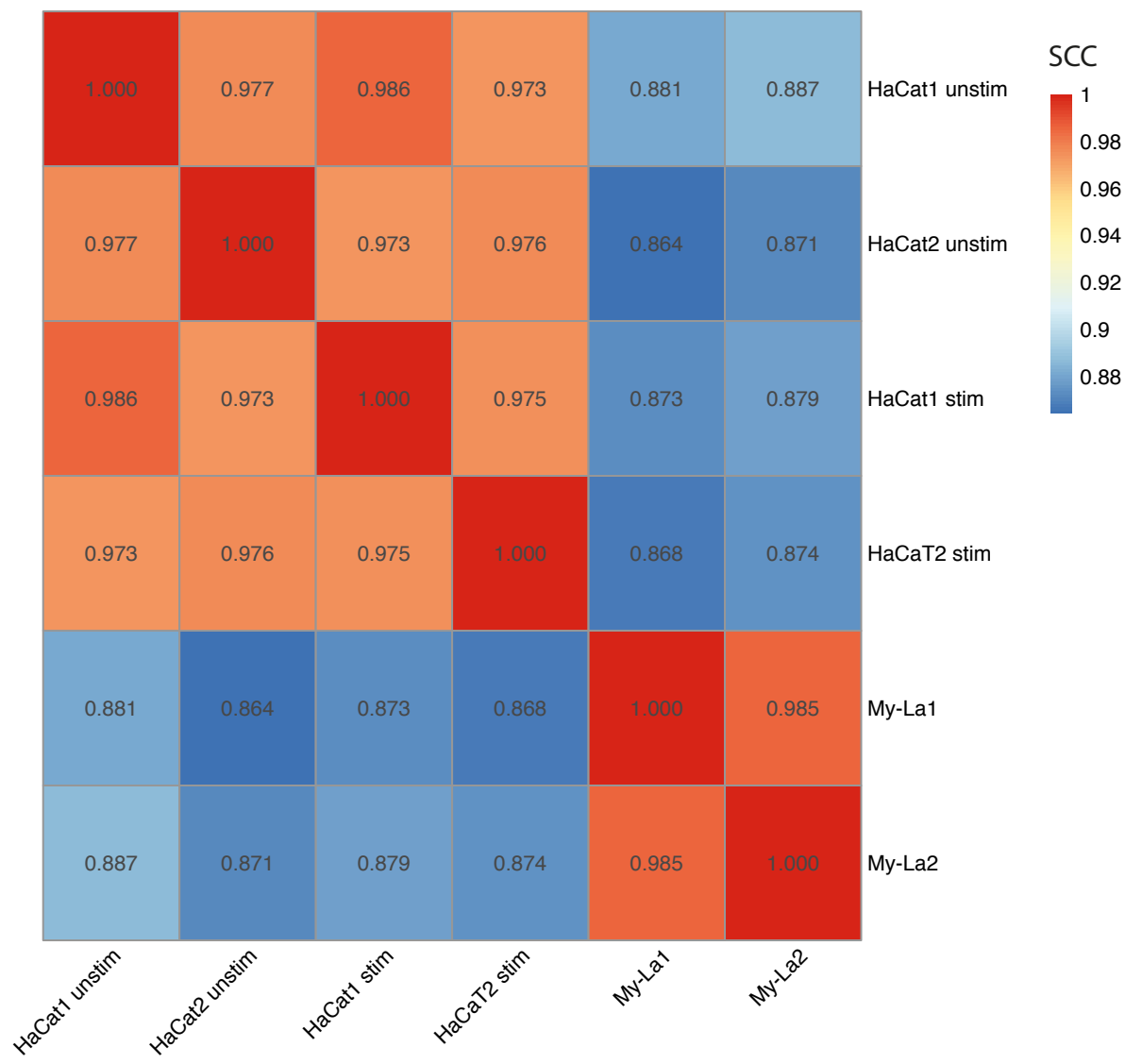

Supplement: Supplementary file 2 — Additional file 2 : Figure S1. Reproducibility between CHi-C replicates for HaCaT (unstimulated), HaCaT (stimulated with IFN-γ), and My-La (down-sampled). A) The number of shared cis-interactions (CHiCAGO score ≥ 5) between replicates are shown in a scaled Venn diagram for each cell type. Each CHi-C replicate was individually analysed by CHiCAGO, as well in the combined analysis where both replicates were submitted to CHiCAGO together. B) Correlation between CHi-C samples was assessed using HiCRep [25] for 10 kb interaction bins on chromosome 1. The heatmap shows the reported stratum-adjusted correlation coefficient (SCC) between samples. [file 12915_2020_779_MOESM2_ESM.pdf]

**HaCaT unstimulated**

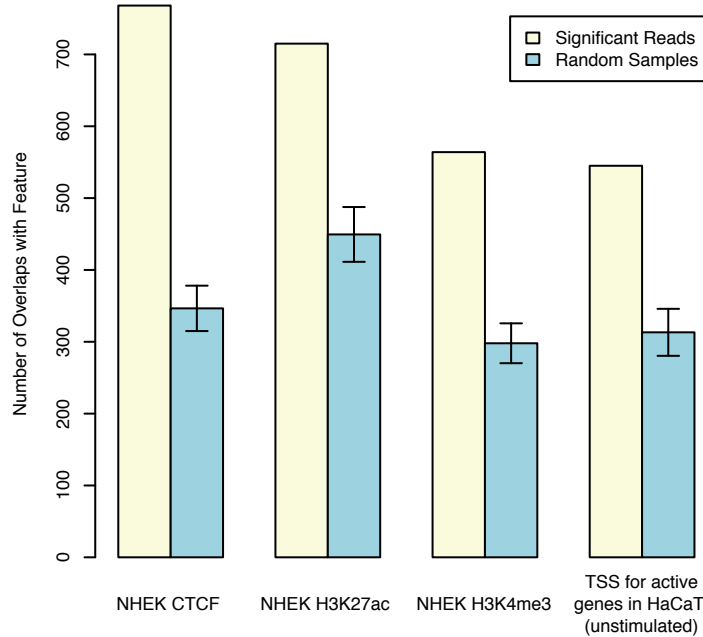

**HaCaT stimulated IFNg**

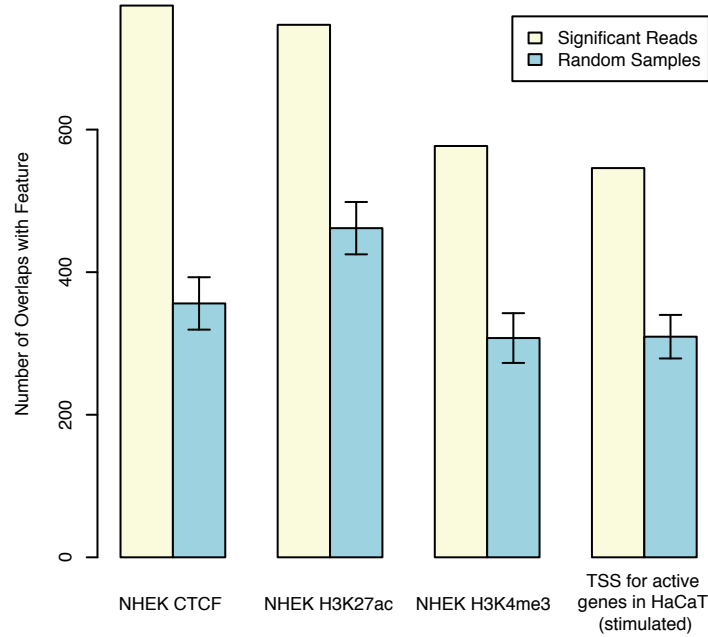

**My-La**

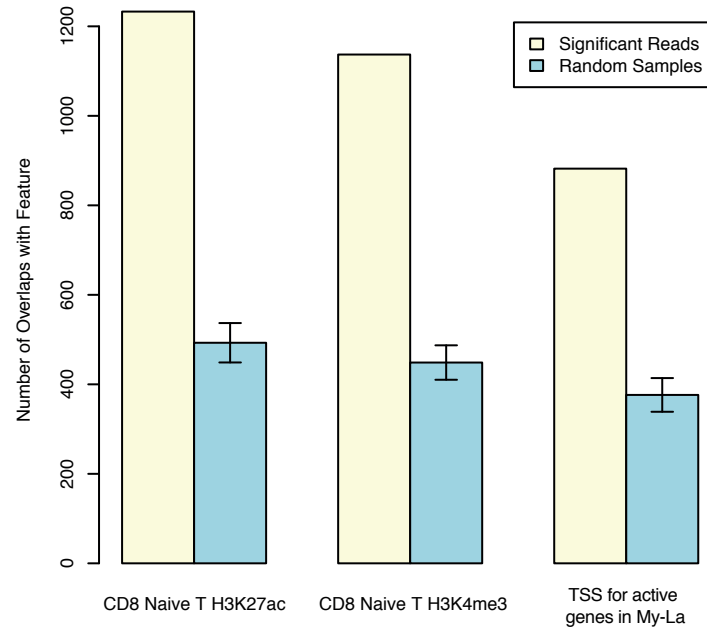

Supplement: Supplementary file 3 — Additional file 3 : Figure S2. Enrichment of features within other-ends of CHi-C interactions. The peak locations of H3K4me3, H3K27ac and CTCF in NHEK (ENCODE), H3K4me3 and H3K27ac in primary CD8+ naïve T cells (Roadmap Epigenomics) [85] and transcription start sites (Ensembl 99) of active genes (read counts > 0) according to the RNA-seq data in HaCaT and My-La cell lines in the present study were tested against other-ends of interactions with all targeted autoimmune loci using the peakEnrichment4Features function of the CHiCAGO package [57]. The graphs show the number of overlaps with the feature in the interaction data (yellow) versus the mean number of overlaps in 100 sampled interactions from the non-significant pool (blue). Error bars show the 95% confidence interval. [file 12915_2020_779_MOESM3_ESM.pdf]

**A**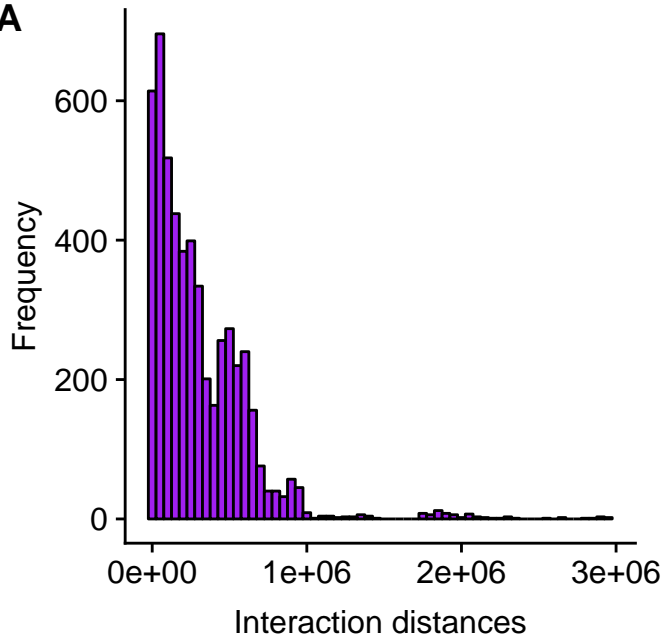**B**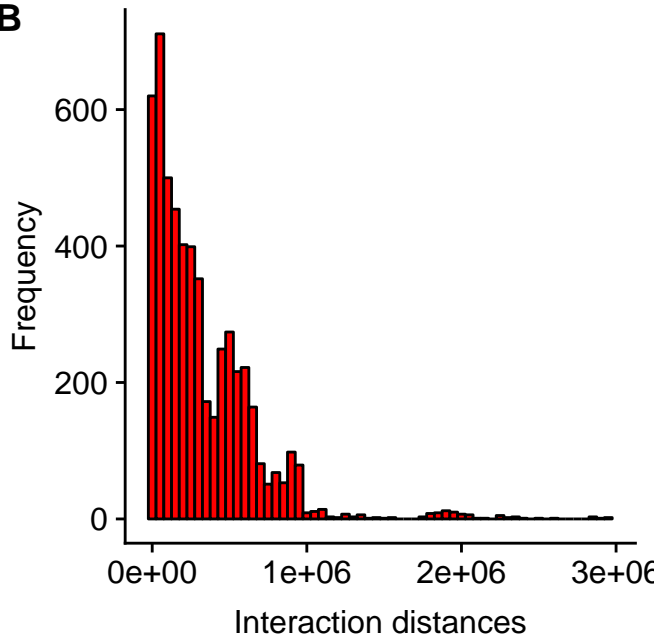**C**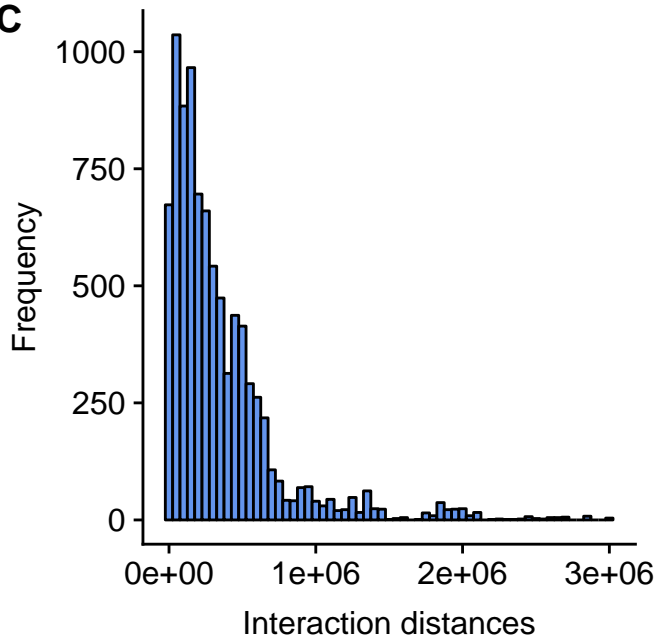

Supplement: Supplementary file 4 — Additional file 4 : Figure S3. Frequency distributions of distances between psoriasis bait fragments and interacting fragments in the CHi-C experiment. The frequency of interactions is shown for 50 kb bins up to 3 Mb in HaCaT unstimulated (A), HaCaT stimulated (B) and My-La cells (C). [file 12915_2020_779_MOESM4_ESM.pdf]

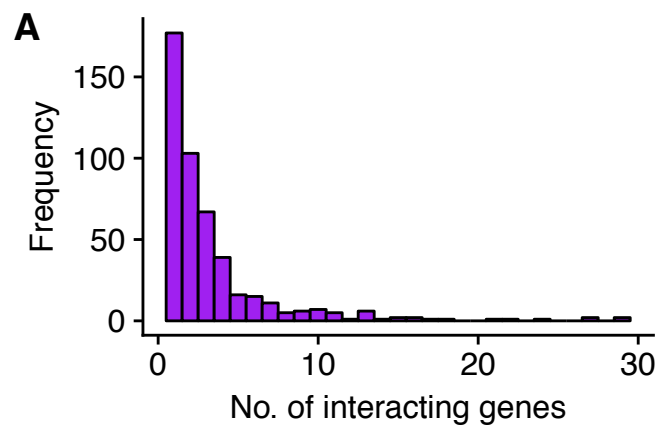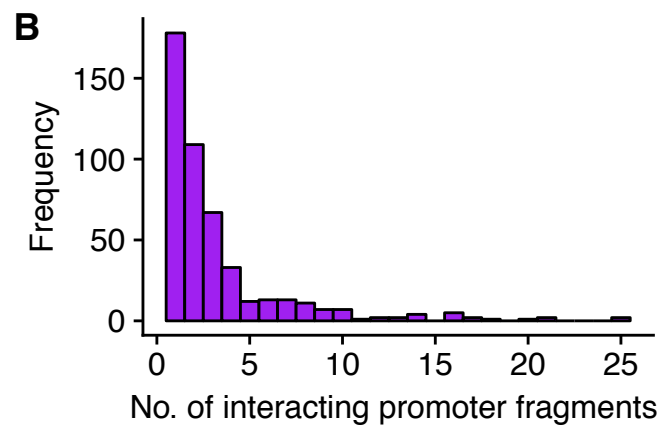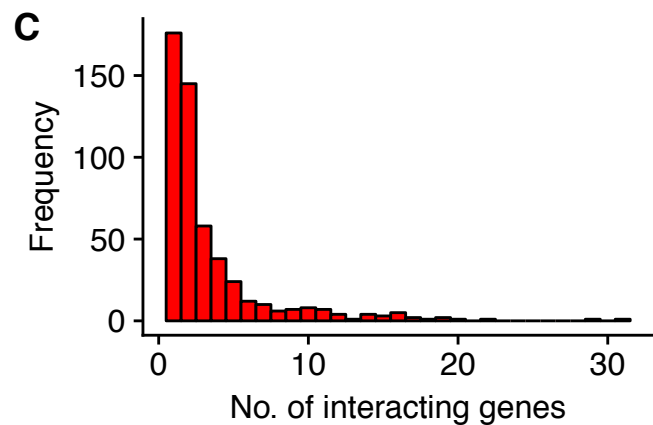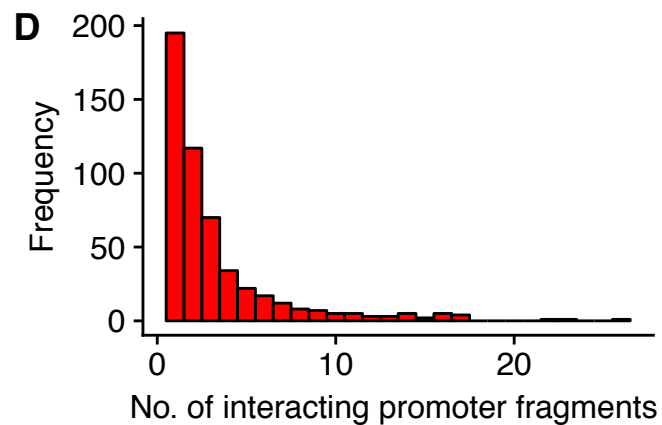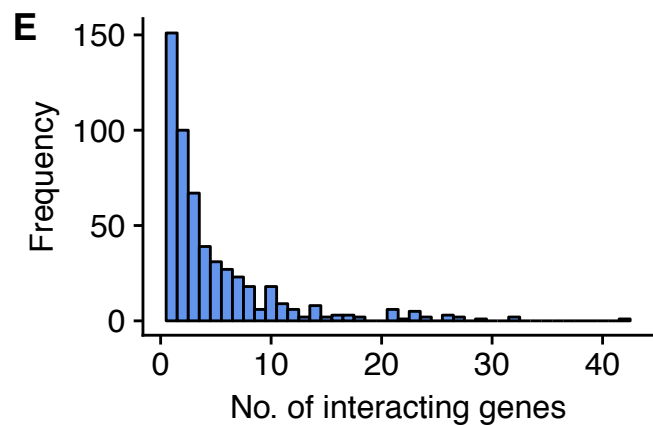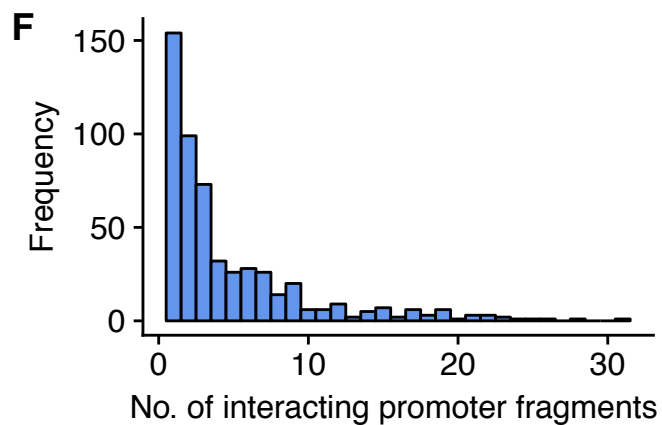

Supplement: Supplementary file 6 — Additional file 6 : Figure S4. Frequency distributions of the number of interactions with promoter fragments per psoriasis-associated bait fragment in the CHi-C experiment. To determine the frequency distribution of psoriasis bait-promoter interactions, the data was firstly restricted to interactions between psoriasis-associated bait fragments and promoter fragments (“Promoter Interactions”). Next, the number of promoter fragments per bait fragment was counted. Of those promoter fragments, the number of corresponding gene promoters was determined. This was necessary because some gene promoters share the same fragment, and some gene promoters are found in more than one fragment. The number of interacting promoter fragments per bait fragment in Promoter Interactions are shown for HaCaT unstimulated (A), HaCaT stimulated (C) and My-La (E). The number of corresponding gene promoters are shown for HaCaT unstimulated (B), HaCaT stimulated (D) and My-La (F). The interaction frequencies are shown in bins of 1. [file 12915_2020_779_MOESM6_ESM.pdf]

HaCaT

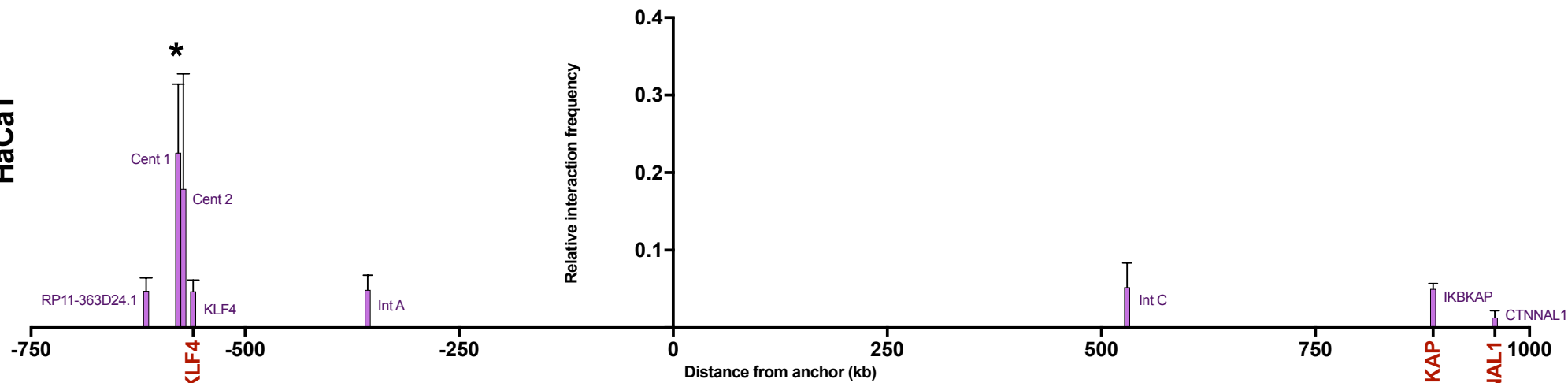

My-La

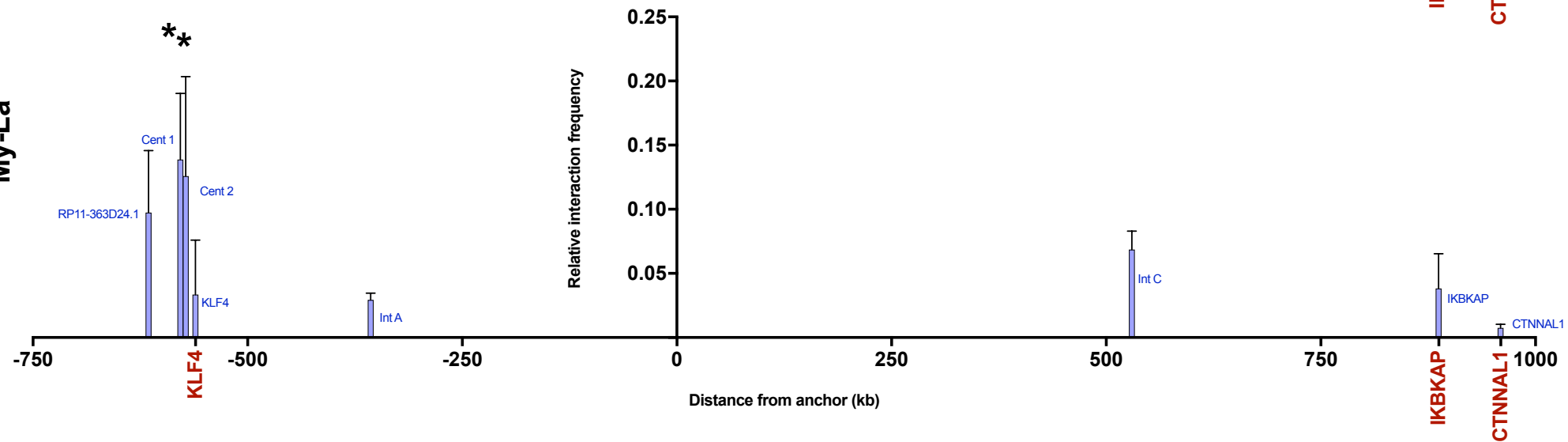

Supplement: Supplementary file 8 — Additional file 8 : Figure S5. 3C-qPCR results in the 9q31.2 locus anchored at the HindIII fragment containing the third psoriasis-associated putative enhancer (rs6477612). qPCR was carried out on HaCaT and My-La 3C libraries using SYBR® Green as the reporter. The anchor fragment at the third psoriasis-associated enhancer is at distance 0 kb. Test fragments were selected in and around KLF4, two points in the gene desert and at fragments containing gene promoters for IKBKAP, FAM206A and CTNNAL1. Interactions were normalised to a short range control. Asterisks denote fragments that had a significantly higher relative interaction frequency than one or more of the other tested fragments, after multiple testing (one-way ANOVA, adjusted P-value < 0.05). Bars show mean + SD of triplicate 3C libraries. Abbreviations: Cent, centromeric; Int, intergenic. [file 12915_2020_779_MOESM8_ESM.pdf]

HaCaT

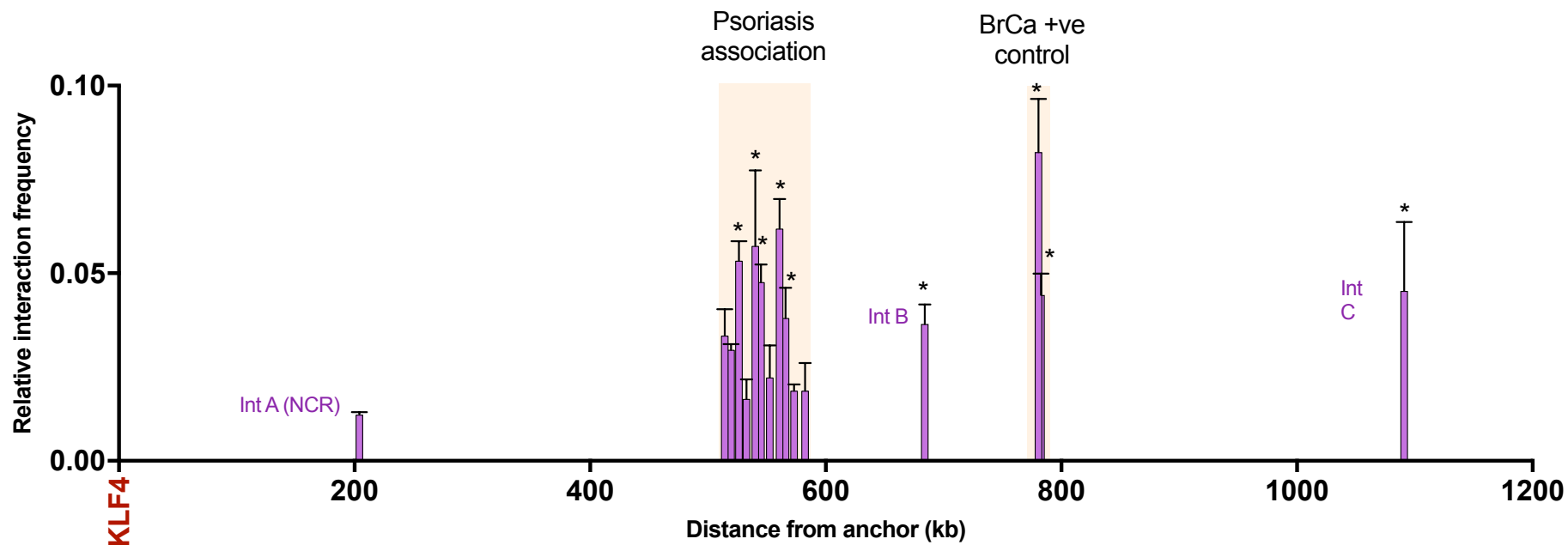

My-Lu

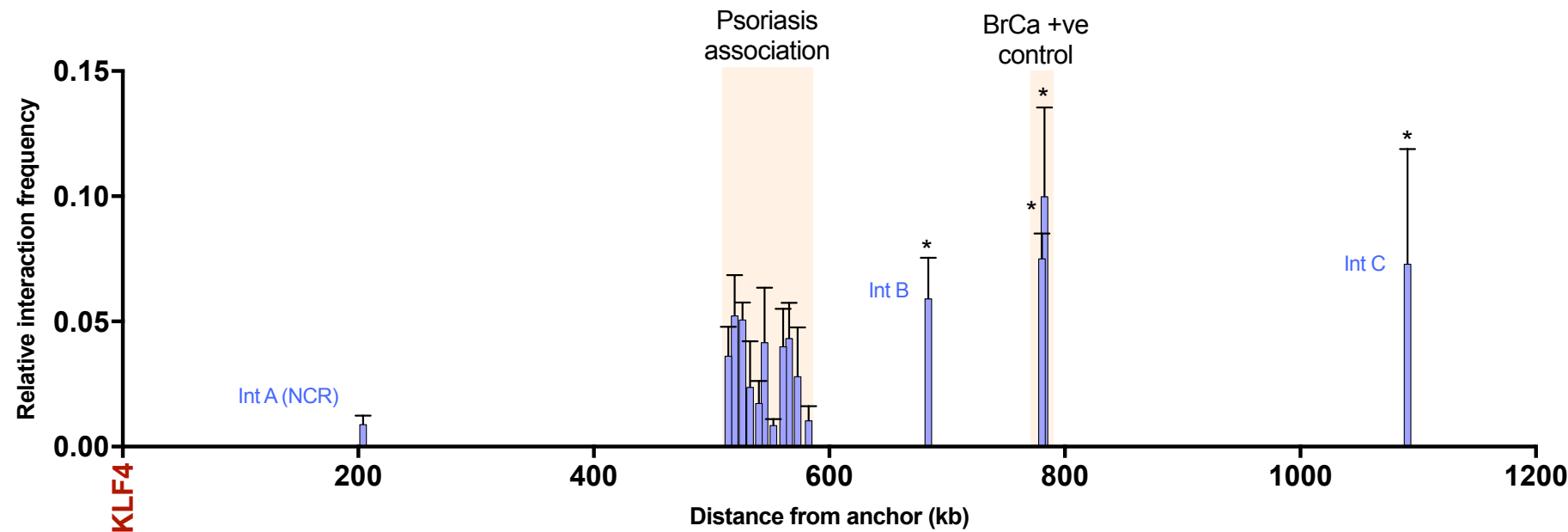

Supplement: Supplementary file 9 — Additional file 9 : Figure S6. 3C-qPCR results in the 9q31.2 locus from the HindIII fragment containing the KLF4 gene and promoter. qPCR was carried out on HaCaT and My-La 3C libraries using TaqMan® as the reporter. The anchor fragment (distance 0) contained the entire KLF4 gene and promoter. An intergenic fragment located approximately 200 kb from the anchor fragment was utilised as a negative control region. Eleven test fragments were selected at regular intervals across the psoriasis association. The positive controls in the Dryden BrCa region were included. Asterisks denote fragments that had a significantly higher relative interaction frequency than the NCR (one-way ANOVA, adjusted P-value < 0.05). Bars show mean + SD of triplicate 3C libraries. Abbreviations: Int, intergenic; NCR, negative control region; BrCa, breast cancer. [file 12915_2020_779_MOESM9_ESM.pdf]

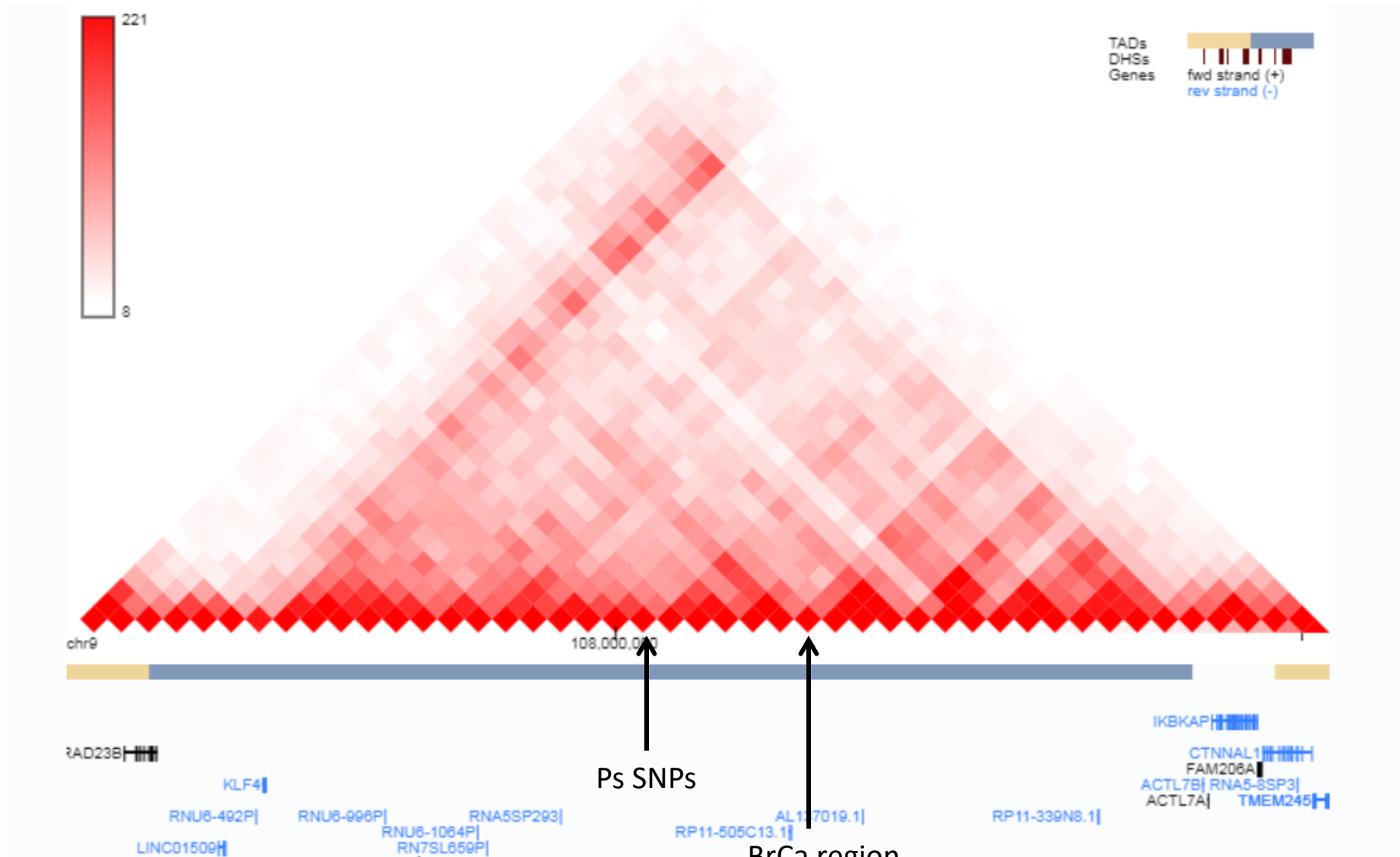

Dryden *et al.*, 2014

Supplement: Supplementary file 10 — Additional file 10 : Figure S7. Previously reported HiC interaction data in NHEK cells in the 9q31.2 locus [14]. Interactions are indicated between KLF4 and the gene desert, including the psoriasis SNPs and the breast cancer region shown in a previous CHi-C experiment [17]. Image created using the YUE lab 3D Genome Browser. [file 12915_2020_779_MOESM10_ESM.pdf]

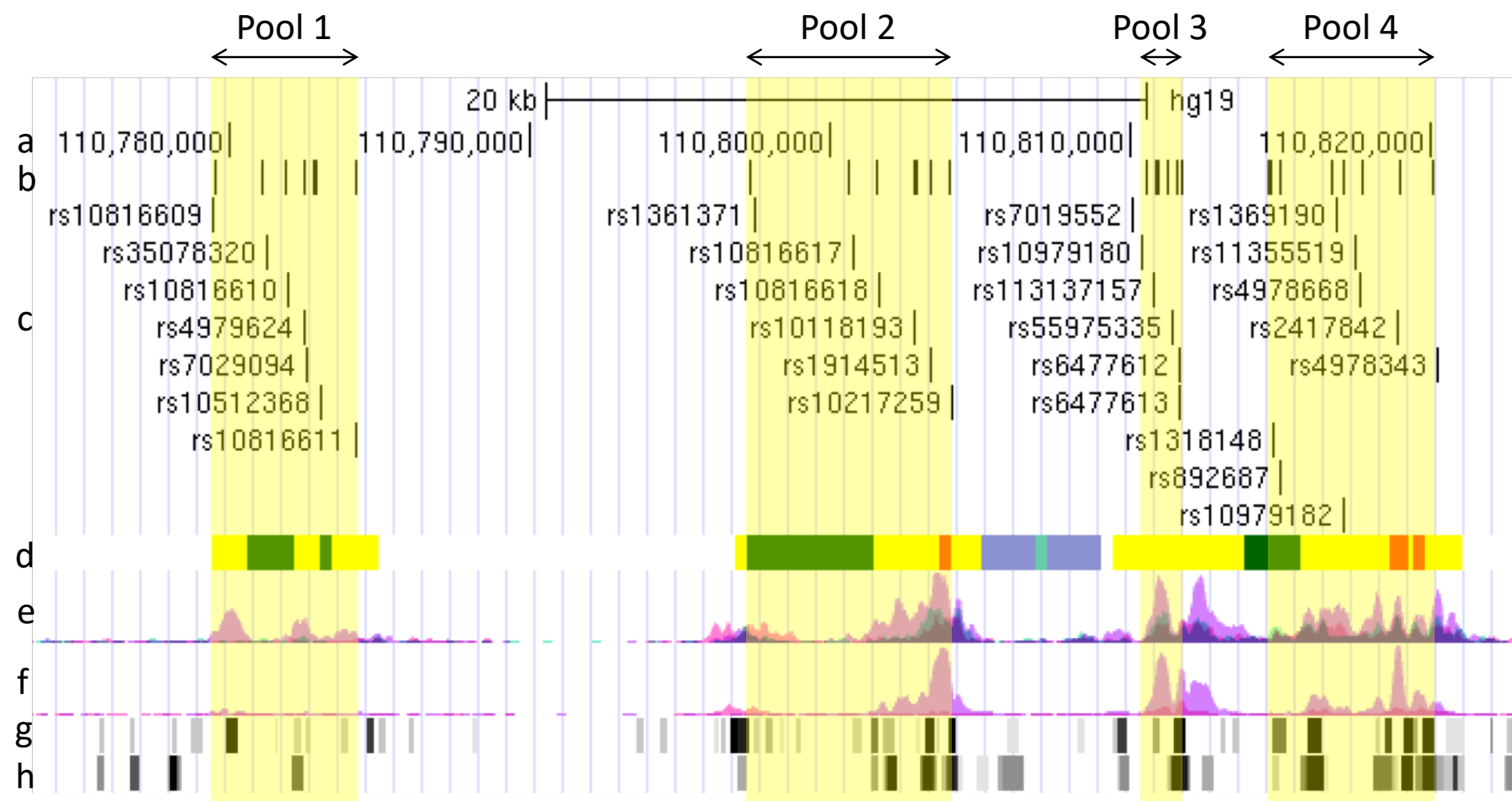

Supplement: Supplementary file 11 — Additional file 11 : Figure S8. sgRNA pools targeting the four putative enhancers in the psoriasis susceptibility locus at 9q31.2. a) location on chromosome 9; b) sgRNA locations; c) SNPs in LD with rs10979182 overlapping putative enhancers; d) ChromHMM segments in NHEK where red, yellow and green indicate “active TSS”, “enhancers” and “transcription” respectively; e) H3K4me1 (ENCODE); f) H3K27ac (ENCODE); g) DNase clusters (ENCODE); h) transcription factor ChIP (ENCODE). [file 12915_2020_779_MOESM11_ESM.pdf]

## IL1RN

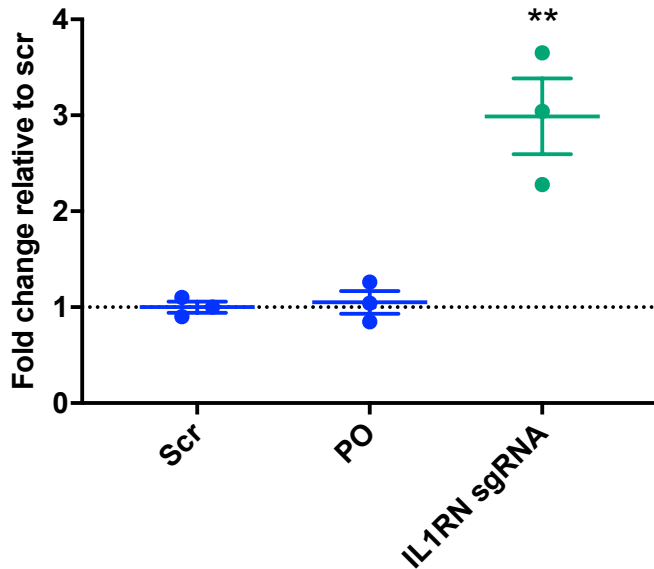

## SLC4A1

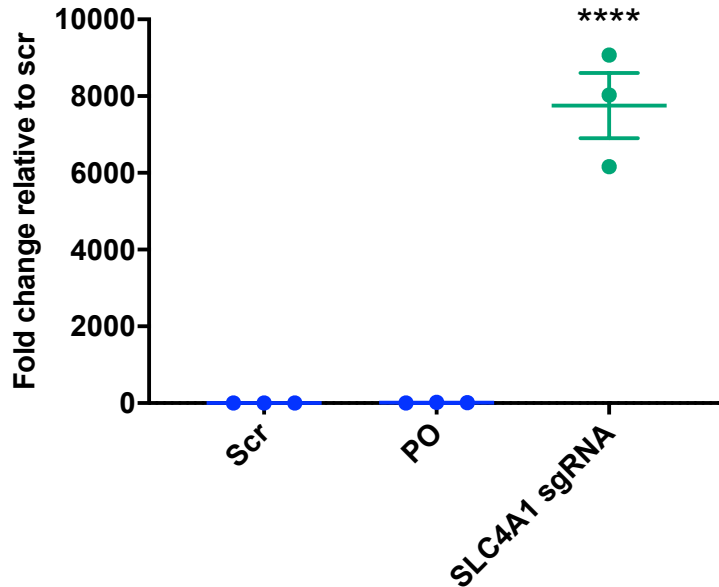

Supplement: Supplementary file 14 — Additional file 14 : Figure S9. Effect of sgRNA targeting IL1RN and SLC4A1 promoters in HaCaT dCas9 P300 cells. HaCaT cells expressing dCas9 P300 were transduced with sgRNA plasmids containing previously-published sgRNA for the IL1RN promoter or the SLC4A1 promoter, in biological triplicate. Control cell lines were generated by transducing HaCaT dCas9 P300 cells with a sgRNA plasmid containing a scrambled guide (Scr) or no guide insert (Plasmid only; PO). qPCR was carried out using TaqMan assays for IL1RN or SLC4A1. Housekeeping genes used were TBP and YWHAZ. Graphs show fold change of gene expression relative to the cells containing the scrambled sgRNA. One-way ANOVA was carried out in GraphPad Prism: in both cases, the cells containing the targeting sgRNA had significantly higher gene expression than the scrambled control (P=0.002 for IL1RN; P=0.0001 for SLC4A1). Asterisks denote P < 0.05. Graphs show the mean fold-change in comparison with scrambled guide, +- SEM of triplicate cell lines. [file 12915_2020_779_MOESM14_ESM.pdf]
