# Supplementary material for: Mapping DNA interaction landscapes in psoriasis susceptibility loci highlights KLF4 as a target gene in 9q31
Source: BMC Biol. 2020 May 4;18:47. doi: 10.1186/s12915-020-00779-3 (PMC7199343; doi:10.1186/s12915-020-00779-3)
Supplement: Supplementary file 13 — Additional file 13 : Table S18. Primers used in the first 9q31.2 3C (SYBR) assay. Table S19. Primers used in the second 9q31.2 3C (TaqMan) assay. Table S20. Primers used in ChIP experiments. Table S21. sgRNA used in the CRISPRa experiment. [file 12915_2020_779_MOESM13_ESM.docx]

Supplementary Methods

Table S18. Fragments targeted in the first 9q31.2 (*KLF4*) 3C-qPCR assay

| Fragment name | Fragment designation | HindIII fragment location (hg19) | Primer sequence |
| --- | --- | --- | --- |
| Ps enhancer 3 (rs6477612) | Anchor | chr9:110810596-110816598 | CAGGGTGTCTAGGAGGTCTTC |
| Enhancer 3 SR | Short-range control | chr9:110824104-110824379 | ACCACATTCCTCTTTTAGCCC |
| RP11-363D24.1 | Target | chr9:110195017-110201199 | AGGAGCTGCATGATTCACCA |
| *KLF4* Centromeric 1 | Target | chr9:110237781-110238441 | GCAGGGCAATGGTCAATTCC |
| *KLF4* Centromeric 2 | Target | chr9:110241992-110244654 | CACACCTAGGAGCCCACAG |
| *KLF4* gene and promoter | Target | chr9:110244654-110255868 | TGCTTTGAAATGAAATCCCTGC |
| Intergenic A | Target | chr9:110452475-110459966 | GGCCAGCTAAGATTCACTGC |
| Intergenic C | Target | chr9:111343493-111346822 | TGAGTACACGCATCTTTTCCT |
| *IKBKAP* & *FAM206A* promoter | Target | chr9:111692307-111703976 | AGCTGTGGTCAATTGGCATT |
| *CTNNAL1* promoter | Target | chr9:111770041-111776135 | GGTGGCGAGGAGAAACAAAA |

Table S19. Fragments targeted in the second 9q31.2 (*KLF4*) 3C-qPCR assay

| Fragment name | Fragment designation | HindIII fragment location | Primer sequence |
| --- | --- | --- | --- |
| *KLF4* gene and promoter | Anchor | chr9:110244654-110255868 | TGCTTTGAAATGAAATCCCTGC |
| *KLF4* gene and promoter | TaqMan probe | chr9:110244654-110255868 | GCTTGCAGCTTTCACAAGGT |
| *KLF4* gene body SR | Short-range control | chr9:110257072-110259991 | GCAAACTCCTCTTATATCCAGGG |
| Intergenic A | Target | chr9:110452475-110459966 | GGCCAGCTAAGATTCACTGC |
| rs10979182 LD region 1 | Target | chr9:110765872-110770045 | TCGAAGACAGGTTGTTGGGA |
| rs10979182 LD region 2 | Target | chr9:110772357-110775650 | CTAAGGCCTGCAATGAAGACA |
| rs10979182 LD region 3 | Target | chr9:110780164-110782236 | GAAGAAGCATCCCATGGCTG |
| rs10979182 LD region 4 | Target | chr9:110783715-110788661 | TAAACCCAAGACAGTGCTGC |
| rs10979182 LD region 5 | Target | chr9:110793130-110796043 | GGCACCTGAGGCATACAATG |
| rs10979182 LD region 6 | Target | chr9:110798743-110801016 | AAATCATGTCCTTGGTGCCC |
| rs10979182 LD region 7 | Target | chr9:110801022-110808470 | TGGCTACATCCAGAGTTGCT |
| rs10979182 LD region 8 | Target | chr9:110810596-110816597 | TCTTCGCTTCCTGTGGGC |
| rs10979182 LD region 9 | Target | chr9:110816603-110821888 | TCAGCTCTTCAAGTTCTCATTCT |
| rs10979182 LD region 10 | Target | chr9:110824384-110829060 | AACCAAGTCACAGAGAAGGCT |
| rs10979182 LD region 11 | Target | chr9:110833319-110838267 | CTAATTGCTGCAGGACCCAC |
| Intergenic B | Target | chr9:110930759-110940017 | CTTGGAGTAGCTTGCTGAGG |
| Positive 1 | Target | chr9:111035783-111036598 | CTCCTCCAATCCCAAGCTGA |
| Positive 2 | Target | chr9:111038412-111038716 | CAGACAAAGACTGGACCCCA |
| Intergenic C | Target | chr9:111343493-111346822 | TGAGTACACGCATCTTTTCCT |

Table S20. Primers used in ChIP experiments

| Target | Primer name | Sequence |
| --- | --- | --- |
| 9q31.2 putative enhancer 1 | 27ac_ChIP_Pool1F1 | CAGAAGCTGTGAGAGGCTGA |
|  | 27ac_ChIP_Pool1R1 | ACTGAGGCAGCCGTAATGTT |
| 9q31.2 putative enhancer 2 | 27ac_ChIP_Pool2F1 | GAGCTGGGGAATGTGTGTTT |
|  | 27ac_ChIP_Pool2R1 | AAATGTGTTTGCCCTGGAAG |
| 9q31.2 putative enhancer 3 | 27ac_ChIP_Pool3F1 | ATCCCTTGTTTAGGGCTTGG |
|  | 27ac_ChIP_Pool3R1 | TGGACCTAGGCTTGCCTCTA |
| 9q31.2 putative enhancer 4 | 27ac_ChIP_Pool4F1 | TGACTGACCCAAGGTCACAT |
|  | 27ac_ChIP_Pool4R1 | GCATATACGGTTTCGGTGTG |
| *KLF4* promoter | CHIP_KLF4_PROM_F1 | CCTGAACCCCAAAGTCAACG |
|  | CHIP_KLF4_PROM_R1 | CGGACCTACTTACTCGCCTT |

Table S21. sgRNA used in the CRISPRa experiment

| Purpose | SNP/gene | Protospacer Sequence |
| --- | --- | --- |
| Scrambled non-targeting control (see Lawhorn et al., 2014) | N/A | AACAGTCGCGTTTGCGACT |
| IL1RN positive control (Perez-Pinera et al., 2013) | IL1RN promoter | CATCAAGTCAGCCATCAGC |
| SLC4A1 positive control (Weissman Lab) | SLC4A1 promoter | GTCAGGAGAACCATGGGGACC |
| 9q31.2 Pool 1 | rs10816609 | CTAATAAGCATCATCGCCCA |
|  | rs35078320 | GTCTCTCTTTAGGCTATCGT |
|  | rs10816610 | GGCTCTTATTCATAGTGTTA |
|  | rs4979624/rs7029094 | AGCTCTTGATATGACCTCAA |
|  | rs10512368 | GCCCAAGACTATGGAATTGT |
|  | rs10816611 | GATAATAGATCTTCCTACAG |
| 9q31.2 Pool 2 | rs1361371 | AAAGTCTAGGTCTCGAATCC |
|  | rs10816617 | GTGAATGCTGATTGTAACCC |
|  | rs10816618 | ATTTATGTATACATCGATTG |
|  | rs10118193 | AGATTCTTGAGAGCGGTAGC |
|  | rs1914513 | CCACACTGCTGCATTGATTC |
|  | rs10217259 | AAGGGGCTAATGCCTGTTCA |
| 9q31.2 Pool 3 | rs10979180 | CCACCAGGGACCCAAAAGGT |
|  | rs113137157 | AATCCCTTGTTTAGGGCTTG |
|  | rs55975335 | CTGAAGGTCCTTTGTTATTG |
|  | rs6477612 | TGGTTTCGAGATTCCTAAAC |
|  | rs6477613 | TGCGTGAGGCTGTACATTAT |
| 9q31.2 Pool 4 | rs1318148 | GATTGGAGACTCGCCATCAG |
|  | rs892687 | CAATAAAAGCCGGGTAGACC |
|  | rs1369190 | AGCTCAGCCTGATTCTCATG |
|  | rs10979182 | GTCAGCCTAGAGGTTTCTAG |
|  | rs4978668 | CGGACTGGTACTTTAGGTGC |
|  | rs2417842 | GGCACAATGCCTTCGAAGGT |
|  | rs4978343 | TTCGTATATCAGTCTTTGCC |
